# Statistical identification of gene association by CID in application of constructing ER regulatory network

**DOI:** 10.1186/1471-2105-10-85

**Published:** 2009-03-17

**Authors:** Li-Yu D Liu, Chien-Yu Chen, Mei-Ju M Chen, Ming-Shian Tsai, Cho-Han S Lee, Tzu L Phang, Li-Yun Chang, Wen-Hung Kuo, Hsiao-Lin Hwa, Huang-Chun Lien, Shih-Ming Jung, Yi-Shing Lin, King-Jen Chang, Fon-Jou Hsieh

**Affiliations:** 1 Department of Agronomy, Biometry Division, National Taiwan University, Taipei, Taiwan; 2 Department of Bio-industrial Mechatronics Engineering, National Taiwan University, Taipei, Taiwan; 3 Graduate Institute of Biomedical Electronics and Bioinformatics, National Taiwan University, Taipei, Taiwan; 4 Department of Clinical Laboratory Sciences and Medical Biotechnology, College of Medicine, National Taiwan University, Taipei, Taiwan; 5 University of Colorado Health Sciences Center, Department of Medicine, Division of Pulmonary Sciences and Critical Care Medicine, Campus Box C-272, 4200 East Ninth Avenue, Denver, Colorado 80262, USA; 6 Department of Obstetrics and Gynecology, College of Medicine, National Taiwan University, Taipei, Taiwan; 7 Department of Surgery, College of Medicine, National Taiwan University, Taipei, Taiwan; 8 Department of Pathology, College of Medicine, National Taiwan University, Taipei, Taiwan; 9 Department of Pathology, Chang Gung Children's Hospital, Taoyuan, Taiwan; 10 Welgene Biotechnology Company, NanGang Business Park, Taipei, Taiwan; 11 Center for Systems biology and Bioinformatics, National Taiwan University, Taipei, Taiwan; 12 Department of Life Science, College of Life Science, National Taiwan University, Taipei, Taiwan

## Abstract

**Background:**

A variety of high-throughput techniques are now available for constructing comprehensive gene regulatory networks in systems biology. In this study, we report a new statistical approach for facilitating *in silico *inference of regulatory network structure. The new measure of association, coefficient of intrinsic dependence (CID), is model-free and can be applied to both continuous and categorical distributions. When given two variables X and Y, CID answers whether Y is dependent on X by examining the conditional distribution of Y given X. In this paper, we apply CID to analyze the regulatory relationships between transcription factors (TFs) (X) and their downstream genes (Y) based on clinical data. More specifically, we use estrogen receptor α (ERα) as the variable X, and the analyses are based on 48 clinical breast cancer gene expression arrays (48A).

**Results:**

The analytical utility of CID was evaluated in comparison with four commonly used statistical methods, Galton-Pearson's correlation coefficient (GPCC), Student's *t*-test (STT), coefficient of determination (CoD), and mutual information (MI). When being compared to GPCC, CoD, and MI, CID reveals its preferential ability to discover the regulatory association where distribution of the mRNA expression levels on X and Y does not fit linear models. On the other hand, when CID is used to measure the association of a continuous variable (Y) against a discrete variable (X), it shows similar performance as compared to STT, and appears to outperform CoD and MI. In addition, this study established a two-layer transcriptional regulatory network to exemplify the usage of CID, in combination with GPCC, in deciphering gene networks based on gene expression profiles from patient arrays.

**Conclusion:**

CID is shown to provide useful information for identifying associations between genes and transcription factors of interest in patient arrays. When coupled with the relationships detected by GPCC, the association predicted by CID are applicable to the construction of transcriptional regulatory networks. This study shows how information from different data sources and learning algorithms can be integrated to investigate whether relevant regulatory mechanisms identified in cell models can also be partially re-identified in clinical samples of breast cancers.

**Availability:**

the implementation of CID in R codes can be freely downloaded from .

## Background

A wide variety of bioinformatics tools are available to assist in studying gene-gene, gene-protein, protein-protein, and protein-metabolite associations that control cellular functions in both prokaryotes and eukaryotes [1,2]. With technologies capable of producing high-throughput data at transcriptomic, proteomic, and metabolomic levels, one has opportunities to accelerate the process of mapping global gene activities into networks and linking them with their corresponding phenotypic features [3-7]. In this study, a novel statistical approach was experimented on human breast cancer gene expression arrays, and the estrogen receptor α (ERα) transcriptional activities were the main focus.

In studies using time course microarray data, correlation analysis continues to serve as one of the most frequently adopted methods in identifying co-expressed gene sets [8-11]. For independent array experiments from patient tissues, association analysis also plays an important role in discovering relationships between transcription factors and their regulated genes [12,13]. It has been shown in those studies that the profile similarities present in co-expressed genes and the association observed in between transcription factors and their direct target genes are usually statistically significant and can be easily detected by correlation measures that aim at identifying linear or partial linear association. However, for the association that cannot fit linear models well, which may be commonly observed in biological systems, less attention has been made due to fewer methods available in measuring such type of association patterns. An alternative approach is to employ non-linear methods that deal with discrete distributions by binning strategy. In this regard, coefficient of determination (CoD) and mutual information (MI) have been proposed to find associated gene pairs [14,15].

Since 2005, a new measure of association, the coefficient of intrinsic dependence (CID), has been introduced to be applicable for microarray analysis in classification and prediction of cancers at molecular level using clinical gene expression arrays [16,17]. CID is designed to uncover the dependency present in between the target (variable Y) and the predictor (variable X) by comparing distributions of the target under different values of the predictor. In this study, CID was further tested in its utility for constructing transcription factor directed regulatory networks using clinical breast cancer gene expression arrays. The statistical analysis conducted in this study reveals the potential of using CID incorporated with correlation test to identify ER-regulated gene sets *in silico *and then to construct a two-layer regulatory network based on clinical breast cancer gene expression arrays.

**Figure 1:**
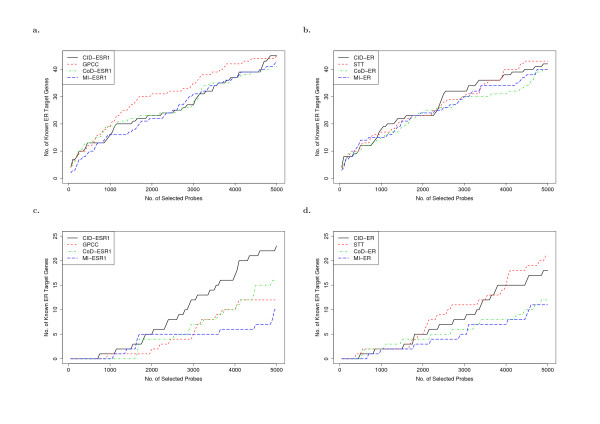
**Number of selected primary target genes of ER (*gene set II*) and non-primary target genes of ER (*gene set III*) versus the number of reported genes that are considered associated with ESR1**. (a) results of using *ESR1 *on *gene set II*; (b) results of using ER+/- on *gene set II*; (c) results of using *ESR1 *on *gene set III*; (d) results of using ER+/- on *gene set III*.

We first use three gene lists to evaluate the power of CID in identifying ER-regulated genes. The first list (*gene set I*) contains a set of genes with expression mechanisms mainly driven by direct binding of ERα to estrogen response element (ERE) in the promoter regions [18,19]. The second and third lists are retrieved from a recent study that provided potential primary (*gene set II*) and secondary target genes (*gene set III*) of ERα based on experiments of a cell culture model MCF-7 [20]. To clarify the contribution of employing CID in detecting ER related genes, we simultaneously include Galton-Pearson's correlation coefficient (GPCC) [9,21,22], Student's t test (STT) [21,23,24], coefficient of determination (CoD) [25-27], and mutual information (MI) [28] when analyzing our patient arrays (48A) with CID. Two types of information are used as the predictor (variable X) when identifying ER-regulated genes. The first one is the mRNA expression level of the gene *ESR1*, and the second one is the protein level status of ERα. In the analysis of using mRNA levels, GPCC shows promising ability of finding ER direct targets (Figure 1a). On the other hand, when applied on discrete variables (ER+/-), CID shows similar performance as compared to STT (Figure 1b), and detects more TF-target associations in *gene set III *than CoD and MI (Figure 1d). Moreover, CID reveals its advantage of discovering indirect or partial linear association on continuous variables (using mRNA levels of *ESR1*) (Figure 1c). This suggests CID's application on construction of large-scale regulatory network, where we can include more functional transcription factors of interest even if their protein level statuses are not experimentally determined.

**Figure 2:**
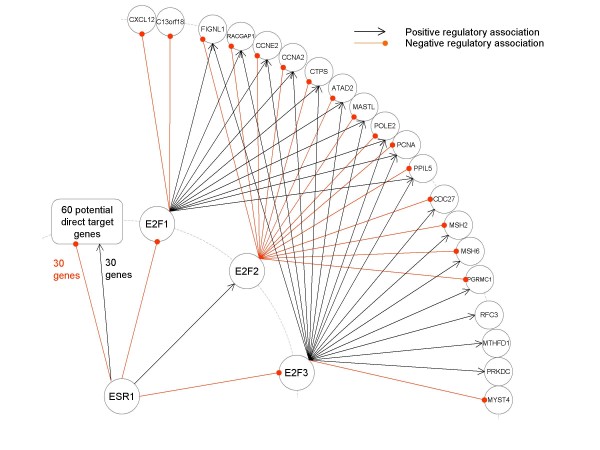
**The established ERα mediated regulatory network is partially constructed by conducting GPCC and CID analyses in 48A**.

In the end, this study shows how information derived from different data sources (a specially conditioned time course data from cell line models and a selected set of independent arrays from patient tissues) and learning algorithms (clustering and various statistical analyses) can be put together to investigate whether the relevant transcriptional regulatory mechanisms built in cell models can be partially re-identified in the given breast cancer systems (Figure 2). Thus, one can attempt to use this knowledge to gain a greater understanding of the breast cancers and uncover ways toward more rational adjuvant hormone therapy for those patients.

## Results and discussion

### Statistical identification of ERα associated genes in 48A

Thirty three genes (*gene set I*) are experimentally proved to have ERE site(s) at the promoter regions by others [18,19]. They are analyzed for the relationships with ERα in our cohort (48A) by different statistical tests. The main focus of this study is to investigate whether the association between a transcription factor (i.e. ERα) and its target genes (e.g. the genes in *gene set I*) can be detected based on expression profiles. In other words, in absence of the protein status, the statistical method is expected to discover the association between the regulators and their targets based only on the mRNA levels of both genes, measured simultaneously in a single experiment. In this regard, statistical methods that deal with continuous variables in both of the conditioners and the targets are the main focus. This includes CID-ESR1, GPCC-ESR1, CoD-ESR1, and MI-ESR1 (see Methods). To show the difference in results when protein level information is adopted, we access the ER status for each patient sample and conduct association analysis by applying statistical methods dealing with discrete variables as the predictors, including CID-ER, STT-ER, CoD-ER, and MI-ER (see Methods). The results are summarized in Tables S2–S4 of Additional File 1.

**Figure 3:**
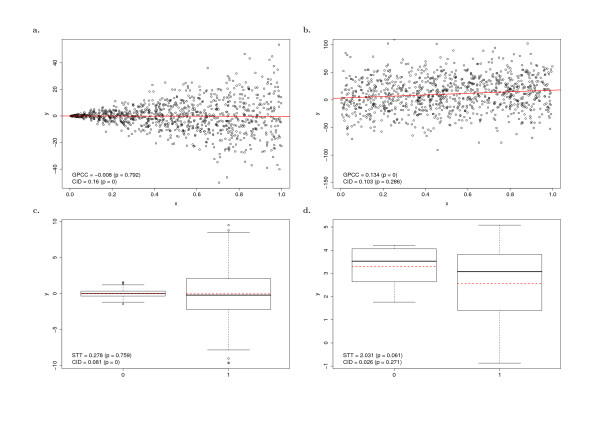
**The brief demonstration of the selected situations when CID, GPCC, and STT evaluate the significance differently based on the same data distribution**. (a)-(b) represent when variables are continuous and thus the CID analysis is compared with GPCC. (c)-(d) represent when one of the variables is discrete and thus the CID analysis is compared with STT.

Here, we first report the results using mRNA levels of *ESR1 *as the variable X. Among the 33 genes analyzed, only four genes (4/33) are consistently detected (*p *≤ 0.05) by all the four tests; 15 genes (15/33) are ranked as significance (*p *≤ 0.05) by at least one of the four statistical tests (Table S2). This indicates that different methods have their preferences in detecting different types of TF-target association patterns based on their gene expression distribution patterns in a given population (Figure 3, Table S1 of Additional File 1, and Additional File 2). Among the 15 genes significantly identified by at least one test, CID-ESR1 claims 12 genes as significance. The same number of genes is identified by GPCC-ESR1. Both CID-ESR1 and GPCC identify more genes than the other two methods. The intersection information between any two methods is summarized in Figure S3a of Additional File 1. Next, for the analyses based on ER status, five genes (5/33) are consistently detected by all the methods; 16 genes (16/33) are ranked as significance by at least one of the four methods (Table S2). In this case, CID-ER identifies the most number of genes (12) among the four tests. Below we use two examples (one is from *gene set I*) to explain why these TF-target gene associations can be discovered by CID-ESR1 and/or CID-ER.

**Figure 4:**
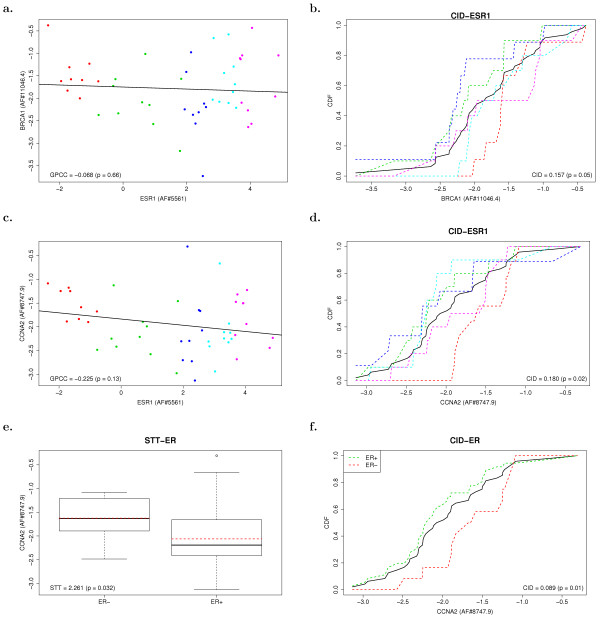
**Statistical analyses for each gene in 48A. Two examples are demonstrated in this figure**. One is *BRCA1 *(a)-(b), which has been significantly identified by CID as the ERα target gene. Another is *CCNA2 *(c)-(f), which was significantly recognized by CID/STT.

CID is designed to measure association between two genes of interest by evaluating the distribution pattern diversity of target gene expressions among patient subgroups, which are partitioned based on the predictor gene expressions in ascending order. Here, two genes, *BRCA1 *(a gene in *gene set I*) and *CCNA2 *(a gene that will be introduced later), are used to illustrate the general interpretations for association measured by CID. The scatter plot of *BRCA1 *versus *ESR1 *mRNA levels is shown in Figure 4a accompanied with the result of GPCC, which is not significant (*p *> 0.05). This plot indicates a mixture of linear and non-linear relationships between *ESR1 *and *BRCA1*. It has been discussed that the promoter region of *BRCA1 *gene might be responsive to estrogen stimulation in both direct and indirect ways [18,19,29]. The indirect model suggests other transcriptional regulators to bind the promoter region before gathering active ERα to form a complex. As the result, it regulates *BRCA1 *expression via either increasing or decreasing mRNA levels in a synergistic manner (i.e. non-linear relationship) [30]. If without the influence from some of regulators, ERα differentially up or down regulates *BRCA1 *mRNA expression via a basal activity of transcriptional mechanism, by which the concentration(s) of all the essential components of transcriptional machine determine the proportional changes of target gene expression levels (i.e. linear relationship). Thus, *BRCA1 *is ERα target gene following both linear and non-linear relationships which were seen by CID (*p *≤ 0.05). CID aims at discovering observations of *BRCA1 *that clustered together given a certain range of expression levels of *ESR1*. Intuitively, if the expression levels of *BRCA1 *are clustered when given low expression of *ESR1*, one yields high prediction power on the expression levels of *BRCA1*. The red points in Figure 4a, for example, indicate one would observe *BRCA1 *having expression level between -2 and -1 with high probability when the expression level of *ESR1 *is within the range (-2, -1). Accordingly, Figure 4b shows the red subgroup contributes the most to the CID value (See Methods).

We further use *CCNA2 *(a gene from the 302 genes mentioned in the next subsection) as an example to illustrate how CID detects remote association between a TF and its target genes. Figure 4c–f describe the association between *CCNA2 *and ERα being evaluated by examining the relative mRNA levels of *CCNA2 *against the mRNA (or protein) levels of *ESR1 *(or ER) in different subgroups. (There are five and two subgroups for CID-ESR1 and CID-ER analyses, respectively.) Through a series of evaluations directed by CID, the clustered subgroup in red is analyzed to be the major contributor (see Methods) to CID-ESR1. STT is designated to measure the difference of means between groups, which are labelled with red dashed lines in Figure 4e. A significant mean difference has been claimed by STT (*p *= 0.032). On the other hand, CID measures not only the difference of means but the diversity of generally distributional patterns. The differential clustering patterns of *CCNA2 *expression in ER(-) as compared to ER(+) is measured by CID-ER with significance (*p *= 0.01).

To further clarify the difference among CID and other statistical methods, we use other two gene lists (*gene set II *and *III*) to demonstrate that different methods may have their own strengths in detecting ERα target genes through primary and non-primary mechanisms. The results have been shown in Table S3 and S4 of Additional File 1. Besides, the intersection information between any two methods is summarized in Figure S3b-c of Additional File 1. In Figure 1, we show the selecting power of these tests by plotting the accumulated number of identified known target genes versus the number of top-ranked genes reported (genes are ranked by the *p*-values in ascending order). Figure 1a shows that GPCC outperforms the other tests in finding ERα direct targets (*gene set II*). However, in Figure 1c, GPCC loses its advantage in detecting ERα regulated genes through non-primary mechanisms (*gene set III*). In Figure 1b, the performance of all the tests is similar (CID-ER and STT-ER perform slightly better than the others). Meanwhile, as shown in Figure 1d, STT demonstrates its ability in detecting ERα non-linear association when provided with ER+/- status. Though CID does not outperform the other methods when using ER+/- status, it is shown in Figure 1c that CID presents its favourable ability of discovering remote association based on continuous mRNA expressions, which reveals its own role in large scale analysis where immunohistochemical status of gene products cannot be always generated.

### Combining CID and GPCC in constructing transcriptional regulatory network

Different statistical methods have their own uniqueness (Table S1 in Additional File 1, Figure 3, and Figure 4), and we have shown in the previous subsection that GPCC has its strength in detecting ERα primary targets. Meanwhile, CID is shown to have preference over GPCC in detecting association between transcription factors and their non-primary downstream genes. While CID shows similar performance with STT in discovering both primary and non-primary association between TFs and the regulated genes when given categorical distributions, CID has the advantage over STT in detecting downstream genes of transcription factors based on only mRNA expression values. This indicates CID to be a new measure of association which has its own role in uncovering TF-target relationships as compared to GPCC and STT. Three methods do share common and different preferences in measuring TF-target association. Thus, we hypothesize that a combined analysis would be desirable for discovering a range of TF-target associations in order to take advantage of the strength from different measures. In this subsection, we use an example to explain how CID can be incorporated with GPCC to discover association between regulators (predictors – variable X) and the regulated genes (targets – variable Y), which have been translated as essential links to build a transcriptional regulatory network. Since protein-level information is not always available in gene expression array analysis, STT is not included in the following example of application.

First, we use time course gene expression profiles on MCF-7 upon estrogen treatment [31] to compile a list of 302 potential estrogen responsive genes by trajectory clustering [32] (see Methods). Among the selected genes, 201 probes (183 genes) were successfully matched in our microarray 48A by gene names. Both GPCC-ESR1 and CID-ESR1 are performed on these 201 candidate probes, resulting in three distinct groups listed in Table 1. The first group contains 60 genes, which are detected by GPCC-ESR1 and thus considered as the potential ER-regulated genes. It is observed that thirteen genes (*FER1L3*, *FKBP4*, *GREB1*, *IL17RB*, *NPY1R*, *PGR*, *PKIB*, *RERG*, *RET*, *RLN2*, *SFXN2*, *SYTL4*, and *TPBG*) in this group is found in *gene set II *(direct target genes of ER predicted in Bourdeau *et al*. [20]). This ratio (13/18) is considerably much higher than random guess (18 60/183 ≈ 5.9 genes).

**Table 1:** Summary for characteristics of the identified gene sets when constructing the two-layer network in Figure 2.

Reported as significance by:	ER direct targets (18^#^)	E2F targets (11^&^)
GPCC-ESR1 (60*)	13	1
CID-ESR1 but not GPCC-ESR1 (22*)	1	7
Neither CID-ESR1 nor GPCC-ESR1 (101*)	4	3

* The number of genes in 48A identified by the statistic of interest.

^# ^The number of genes in 48A appeared in *gene set II*.

^&^The number of genes in 48A appeared in the gene list of E2F family direct target by others [38].

The 22 genes in the second group shown in Table 1 are reported to be significantly associated with the mRNA levels of ESR1 by CID-ESR1, but not by GPCC-ESR1. It is observed that some of the genes in this group are target genes of E2F family members. For example, *CCNE2 *and *PCNA *are previously reported to be regulated by *E2F1*, and *CCNA2 *is found to be mainly targeted by E2F family but is also as one of ERα target genes [33-37]. In this regard, we apply a further GPCC test on those 22 genes versus each of the E2F family members appeared in our microarray, including one probe of *E2F1*, *E2F2*, *E2F4*, *E2F5*, *E2F6*, and *E2F7 *and two probes of *E2F3*. In addition, GPCC-ESR1 is performed on each E2F member to validate the association between *ESR1 *and E2F members in our array data (48A). As shown in Figure 2, only three activators *E2F1*, *E2F2*, and one probe of *E2F3 *show significant dependency on ESR1 by GPCC in our cohort. Among the 22 genes in the second group, 20 of them (20/22) are found significantly correlated with at least one of the expression levels of *E2F1*, *E2F2*, or *E2F3*.

It is worthy of notice that some of essential relationships in a transcriptional regulatory network in general [38-40] are found in Figure 2. For instance, nine of them (*ATAD2, CCNA2*, *CCNE2*, *MSH2*, *MSH6*, *PCNA*, *PRKDC*, *RACGAP1*, and *RFC3*) have been reported as E2F target genes in another study [29,38], and *FIGNL1 *is predicted as a novel E2F1-inducible gene in [39]. Among the 22 genes in this group, only two of them (*NRCAM*, and *C9orf80*) do not show their dependency with any E2F members, and thus are not shown in Figure 2.

We wonder if we can conclude that most genes in the first group in Table 1 are estrogen responsive genes through the primary mechanism and the second group through non-primary mechanism involving other regulators. As discussed previously, among the 18 genes of *gene set II*, 13 genes are found in the first group, but only one gene is found in the second group. Furthermore, we observe that, among the tested 183 genes, 11 of them are E2F target genes reported in [38]. As shown in Table 1, seven of the 11 E2F target genes fall in the second group, but only one is found in the first group. The differential characteristics of the first group (60 genes) and the second group (22 genes) reveal the advantage of incorporating CID with GPCC in constructing regulatory network. Finally, it is observed that some previously annotated relationships (four ER direct targets and three E2F targets) [20,38] are falling in the third group (101 genes). Thus, they are speculated as not being significant in our cohort. Some gene expression relationships in Figure 2 are unknown relationships and deserve to be further investigated by *in vitro *studies.

Interestingly, *CCNA2, CCNE2*, and *PCNA *show being down-regulated in our breast cancer cohort. It indicates the suppressive expression of those identified genes regulated by ERα mediated transcriptional activities, which is opposite to that in the estrogen treated MCF-7 model [31,36]. It has been discussed previously that ERα transcriptionally regulates *E2F1 *expression via indirect tethering mechanism [33]. In the presence of estrogen, *E2F1 *is the major transcriptional regulator and/or the co-regulator of genes mediating cell cycle *in vitro *[36]. Therefore, we reason that upon estrogen exposure ERα suppressed *E2F1 *mRNA expression in our cohort. The research evidence also support E2F1 may being a major transcription factor of *CCNA2, CCNE2 *and *PCNA *upon estrogen exposure [36,37,41]. As a consequence of ERα suppressive effect on the gene expression of *E2F1 *in ER(+) population of 48A, we conclude that ERα suppresses the mRNA expression of *CCNA2, CCNE2*, and *PCNA *mainly via *E2F1*, at least in part.

One drawback for CID is that it does not tell whether it is positive or negative association when a subject gene is considered statistical dependent to the query transcription factor. In this regard, GPCC is suggested to supply the required information. Finally, we conclude that the example shown in Figure 2 reveals the possibility of efficiently constructing regulatory network for scientists to generate more hypotheses based on statistical tests. In this paper, we consider only one regulator X at a time to examine whether it is related to the expression levels of the regulated gene Y. In molecular systems, however, it is commonly observed that multiple regulators (multiple X's) simultaneously govern the behaviour of Y. By definition, CID is ready to be extended to measure associations between multiple predictors (X's) and the target (Y). How to construct a more realistic network by integrating such multivariate associations identified by CID deserves further studies.

## Conclusion

We have developed a methodology for extracting a transcriptional regulatory network in a high-throughput gene expression data set. First, a new measure of association CID is demonstrated to provide additional information to other traditional tests. Second, a small example is employed to illustrate that how estrogen responsive genes with similar expression profiles can be first retrieved based on time course experiments and then the structure of network can be discovered by association analysis combing GPCC and CID. We conclude this statistical approach to be novel and it facilitates the process of drawing a statistically relevant network in a given population.

## Methods

### Clinical breast cancer expression array

All the 48 clinical arrays (48A) used in this study were from a patient cohort (started from 2002 to 2005) collected at National Taiwan University Hospital (NTUH). The tumor samples were defined by greater than 50% tumor cells per high-power field examined in a section adjacent to the tissue used. These clinical arrays were generated using the Human 1A (version 2) oligonucleotide microarray from Agilent technologies, according to the methods provided by the manufacturer [42]. All patients had given informed consent according to guidelines approved by the Institutional Review Board (IRB) of NTUH. The quality control of expression arrays was verified by quantitative measurement of the mRNA levels of four chosen genes, which each was normalized by the constitutive mRNA expression level of TATA box-binding protein (*TBP*). Estrogen receptor α (*ESR1*), progesterone receptor A (*PGR*), G protein coupled receptor 30 (*GPR30*), and human epidermal growth factor receptor-2 (*HER-2/neu*) were the selected four genes. The data was generated via quantitative reverse transcriptase polymerase chain reaction (qPCR) and the detailed procedure was described previously [43]. Four linear correlation plots, showing the consistency between array and qPCR measurements are in Additional File 1 (Figure S1), demonstrating the quality control (QC) data for 48A.

### Immunochemical staining of ERα

All the paraffin sections of breast cancer specimens (3–5 μm in thickness) on slides were processed in Ventana's automated staining system (BenchMark^® ^LT) (Ventana Medical Systems, Inc) for the immunohistochemical stain (IHC). There were two main steps. Firstly, the slides were probed with CONFIRM™ anti-Estrogen Receptor (SP1) rabbit monoclonal primary antibody (Catalog # 790–4325, Ventana Medical System Inc., Tucson, AZ, USA). Secondly, to localize and visualize ERα protein within the specimen, iVIEW™ DAB Detection kit (Catalog # 760-091, Ventana Medical System Inc.) was applied. The negative control slides for tumor specimens were solely stained using iVIEW™ DAB Detection kit (Catalog # 760-091, Ventana Medical System Inc.). All the slides after immunostain were further examined by two experienced pathologists. There are 12 ER(-) and 36 ER(+) specimens in 48A. Based on qPCR, the *ESR1 *mRNA levels (-ΔC_p_) were ranged from -4 to 3 for ER(+) group and from -9 to -4 for ER(-) group (Figure S2 in Additional File 1).

Jarzabek *et al*. [44] reported that the lack of ERα protein expression is not due to lack of ERα gene expression or methylation of ERα promoter, but due to differential post-transcriptional or post-translational mechanisms. In addition, Potemski *et al*. [45] reported their results not supporting ER mRNA to be a key factor in molecular distinction between breast tumors. However, we found *ESR1 *(AF#5561, AF# stands for Agilent feature number) and IHC of ER status is positively correlated but not in a linear relationship (data not shown). Poola and Yue [46] suggested a clinical applicable approach in using ERα mRNA level as the quantitative analysis for identification of ER(+) breast cancer. And, one should be noticed that the definition of positive IHC stain for ERα protein in this study is for tumor slide having shown greater than 10% tumor cells with moderate to high amount of immunoreactive nuclear ERα protein. In this study, we first used IHC and qPCR data to demonstrate that the immunohistochemical status of ERα is correlated to its mRNA levels. After that, we adopted the data in Figure S1 to illustrate the validity of using array data for large-scale association analyses in this study.

### Microarray preprocessing and statistical analyses

Microarray raw data were through data processing which included background correction, elimination of poor quality spots, and log transformation of RNA measures relative to a reference (Stratagene's human common reference RNA) using base-2 logarithm (Detailed information about the gene expression data can be found at ).

### Coefficient of intrinsic dependence

The main statistical method applied in this paper for identifying the gene lists of estrogen regulated transcription activities was the coefficient of intrinsic dependence (CID) [16] with a few modifications. This new measure is model-free and can handle both continuous and categorical variables. For the genes of interest, we employed CID to measure their individual association with both ERα IHC status (ER) and ERα mRNA status (*ESR1*) and compare the results with other applicable statistical methods in either case.

CID-ESR1 is designated to describe the association between *ESR1 *and a gene of interest. The computation of CID-ESR1 for a selected gene includes several phases. First, CID promoted subgrouping the entire cohort (48A) into five approximately equally sized subgroups. The rationale of dividing the cohort as five subgroups was aiming at preserving the minimum number in each subgroup (≈ 10) required for meeting the statistical accuracy of CID analysis [16]. The partition of those five subgroups was based on their presorted mRNA expression levels of *ESR1 *in an ascending order. The smallest 20% of mRNA expression levels of *ESR1 *were assigned to subgroup 1; the smallest 20–40% of mRNA expression levels of *ESR1 *were assigned to subgroup 2; and so on. In Figure 4a and Figure 4c, the five subgroups constructed according to the ascending order of *ESR1 *mRNA levels were marked with different colors. Let symbol *y*_*i *_and *x*_*i *_denote the mRNA levels of gene Y and *ESR1 *for the *i*-th individual, respectively. In each subgroup *j*, the following quantity was evaluated:

(1) $$\sum_{i=1}^{N}{\left[F_{N_{j}}(y_{i})-F_{N}(y_{i})\right]}^{2},$$

where

$$\begin{array}{c} F_{N}(y_{i})=\frac{1}{N}\sum_{k=1}^{N}I(y_{k}<y_{i}),\\ F_{N_{j}}(y_{i})=\frac{1}{N_{j}}\sum_{k=1}^{N}I(y_{k}<y_{i}\text{ and }x_{k}\in \text{the }j\text{-th subgroup}),\\ I(A)=\{\begin{array}{ll} 1, & \text{if }A\text{ is true};\\ 0, & \text{if }A\text{ is not true}; \end{array}\\ N_{j}\text{ is the size of }j\text{-th subgroup},\text{and}\\ N\text{ is the total number of samples}\text{.} \end{array}$$

In the case studied here, *N *= 48 and *N*_*j *_= 9 or 10. The quantity in Equation (1) could be visualized in Figure 4b and Figure 4d. The black solid curve, representing *F*_*N*_(*y*_*i*_), is called empirical cumulative distribution function (CDF) of Y, which is evaluated at all possible values within the range of mRNA levels of gene Y. The colored curves, representing $F_{N_{j}}$(*y*_*i*_), are conditional CDF's of Y for the corresponding subgroups in Figure 4a and Figure 4c. The discrepancy between *F*_*N*_(*y*_*i*_) and (*y*_*i*_) measures the levels of dependence within the subgroup. A weighted average is taken to account all discrepancies measured within different subgroups and yields the value of CID-ESR1:

$$\text{CID-ESR}1\text{ for gene Y}=\frac{1}{C(N)}\sum_{j=1}^{K}\frac{N_{j}}{N^{2}}\sum_{i=1}^{N}{\left[F_{N_{j}}(y_{i})-F_{N}(y_{i})\right]}^{2},$$

where *K *= 5 is the number of subgroups and *C*(*N*) is a constant depending only *on N *to ensure the CID values are within the range [0,1] [17]. In particular, *C*(*N*) = 1/6 - 1/[6(*N*)^2^] if the Y variable is continuous with all distinct values. In the case studied here, *C*(*N*) = 0.1666 = 1/6 - 1/[6(48)^2^]. CID = 0 stands for "Independent" and CID = 1 for "Fully dependent".

CID can also evaluate the differential expression patterns of genes in between ER(+) and ER(-) clinical arrays. We designate it as CID-ER. The entire cohort (48A) was divided into two subgroups, ER(+) and ER(-), respectively. The computation of CID-ER is similar with that of CID-ESR1, except the cohort (48A) was divided into two subgroups (ER(+) and ER(-)) instead of five. Figure 4f provided one example of the computation of CID-ER. The black solid curve represents the empirical CDF of Y, while two colored curves represent empirical conditional CDF's of Y for corresponding subgroups ER(+) (green) and ER(-) (red), respectively. CID-ER can be computed by

$$\text{CID-ER for gene Y}=\frac{1}{C(N)}\sum_{j=1}^{2}\frac{N_{j}}{N^{2}}\sum_{i=1}^{N}{\left[F_{N_{j}}(y_{i})-F_{N}(y_{i})\right]}^{2},$$

where *N*_*j *_= 36 and 12 for ER(+) and ER(-) subgroups, respectively, and *C*(*N*) = 0.1666 like that in CID-ESR1.

The subgroup of gene Y whose conditional CDF was the farthest away from the CDF of the whole cohort (black solid line) (for example, the red dashed line in Figure 4b, the light-blue dashed line in Figure 4d and the red dashed line in Figure 4f) contribute as the largest to the CID value of gene Y and resulted in the significance evaluated by CID [17]. It can be observed that the subgroups contribute the most to the CID values are also the most aggregated subgroups in the scatter plots.

### Other statistical methods for comparison

Four more statistical methods were included in this study in addition to CID. They were Galton-Pearson's correlation coefficient (abbreviated to GPCC; analysis for continuous variables) [9,21,22], Student's *t*-test (abbreviated to STT; analysis for binary variables) [21,23,24], coefficient of determination (CoD) [25-27], and mutual information (MI) [28]. CoD and MI were applied to measure association of genes with both ERα IHC status (ER) and ERα mRNA status (*ESR1*). However, CoD and MI are originally designed for discrete data only (can be either binary or multiple classes) and partitioning is required in order to account for association between continuous variables. We intuitively partitioned the entire cohort (48A) by the same method used by CID. The brief feature descriptions for these statistical methods are demonstrated in Table S1 of Additional File 1. CID consists of the concepts for using the statistical evaluations on the significance for the linear and nonlinear association between two genes of interest and describes the significance by evaluating the distribution pattern of gene expression in subgroups. When two genes have the linear association, it indicates the expression profiles of those two genes being proportional or inversely proportional to each other. Otherwise, we claimed that two genes have nonlinear association.

In Figure 3, two typical distinguished features of CID are demonstrated in comparison with two most commonly used methods, GPCC and STT. When the expression values of gene Y under a given expression condition of gene X are clustered together, CID gives a higher score indicating the significance in association but no significance observed by GPCC (Figure 3a). However, when the scattering expression pattern occurs, CID gives a lower score indicating independent association (Figure 3b). In the case of Figure 3b, the data is considered to be insignificant by CID analysis but it is found to be significant by GPCC as linear association (Figure 3b). When the binary variables (e.g. ER+/-) are applied in finding the association patterns, STT only evaluates the significance statistically by evaluating whether two sample means are different or not regardless the data distribution patterns (Figure 3d), while CID determines the significance solely via the closely clustered distribution of data even both variables having the similar means (Figure 3c).

### Accessing the significance of genes by permutation

After obtaining a statistic for gene *Y*, the *p*-values of the statistics were accessed by 1,000 times of permutation. In each time of permutation, the 48 mRNA levels of gene *Y *are randomly reordered. The statistics can be computed again based on reordered mRNA levels of gene Y. These 1,000 values of statistic obtained by random permutations mimic the distribution of the statistic under independence. The *p*-value is accessed by the number of 1,000 simulated values greater than or equal to the observed value of statistic divided by 1,001. The genes were ranked according to the ascending order of *p*-values. Whenever there are more than one gene relate to the same *p*-value, the ranking scores for those genes are given differently based on their observed values of statistics in a descending order. The permutation procedure described above is applied to all the statistical tests conducted in this paper.

### Gene lists for performance comparison

Three lists are collected for comparing the predicting power of CID with GPCC and STT. The first gene list contains 33 genes that were characterized as ERα target genes via the *in vitro *findings of both O' Lone *et al*. [19] and Klinge [18]. These 33 genes have ERE at their promoter regions. This gene set is designated as *gene set I *throughout the paper. In addition to *gene set I*, we further include the primary and secondary estrogen target genes reported in a recent study [20]; two lists are provided in its Table 1, where the list on the right-hand side provides the potential primary genes regulated by estrogen and the un-bolded genes on the left-hand side are considered as potential secondary (or even higher order) target genes. We organized these two gene lists as *gene set II *(direct target genes of ER) and *gene set III *(indirect target genes of ER) by finding the corresponding gene probes in our arrays, which results in 85 genes in *gene set II *and 46 genes in *gene set III*.

### MCF-7 time course expression array

The MCF-7 time course expression array (Affymetrix human genome u133 plus 2 arrays) was downloaded from supplementary data in online publication of Carroll *et al*. [31]. The data contains gene expression profiles of MCF-7 upon estrogen treatment for 4, 8 and 12 hours, respectively. After gene filtering and statistical test (ANOVA), there were 1,438 genes left. Those genes were then feed into the trajectory clustering algorithm [32]. In total, 302 genes were classified as continuously up-regulated estrogen responsive genes after trajectory clustering (the increase-increase-increase (III) pattern). In this paper we used these 302 genes as a gene set for constructing a two-layer regulatory network. After comparing the gene symbols of the 302 genes with the probe list of our arrays, total only 183 genes (represented in 201 probes) are known for their gene names. Therefore, they are collected as the gene candidates of the constructed network.

## Authors' contributions

Both LYDL and CYC initiated the study. LYDL took the major credit in conducting the entire statistical approach. TLP provided the list of 302 genes based on applying trajectory clustering on a given data set. MJMC, MST, and CHSL helped collecting references and were working under supervision in early development of this methodology. HLH, WHK, and KJC provided with the clinical pathological information for this study. FJH provided the samples, clinical data, and major funding for the experiments. LYC joined the data translation from statistical side to biological side with LYDL and CYC. LYDL, LYC, and CYC wrote the manuscript. YSL provided data of 48 clinical gene expression arrays. HCL and SMJ consulted for the results from immunohistochemical staining. All authors have read and approved the final manuscript.

## Supplementary Material

Additional file 1 **Supporting results of statistical analyses.** This file has detailed information for statistical methods as well as the supplementary figures, tables, text and references. Click here for file

Additional file 2 **Plot analyses for gene set I.** This file contains graphical illustration of statistical analyses for those genes appeared in gene set I. Four plots for each gene represent four tests (CID-ESR1, CID-ER, GPCC, and STT), respectively. Click here for file
