# Supplementary material for: Statistical identification of gene association by CID in application of constructing ER regulatory network
Source: BMC Bioinformatics. 2009 Mar 17;10:85. doi: 10.1186/1471-2105-10-85 (PMC2679734; doi:10.1186/1471-2105-10-85)
Supplement: Additional file 1 — Supporting results of statistical analyses. This file has detailed information for statistical methods as well as the supplementary figures, tables, text and references. [file 1471-2105-10-85-S1.pdf]

Figures:

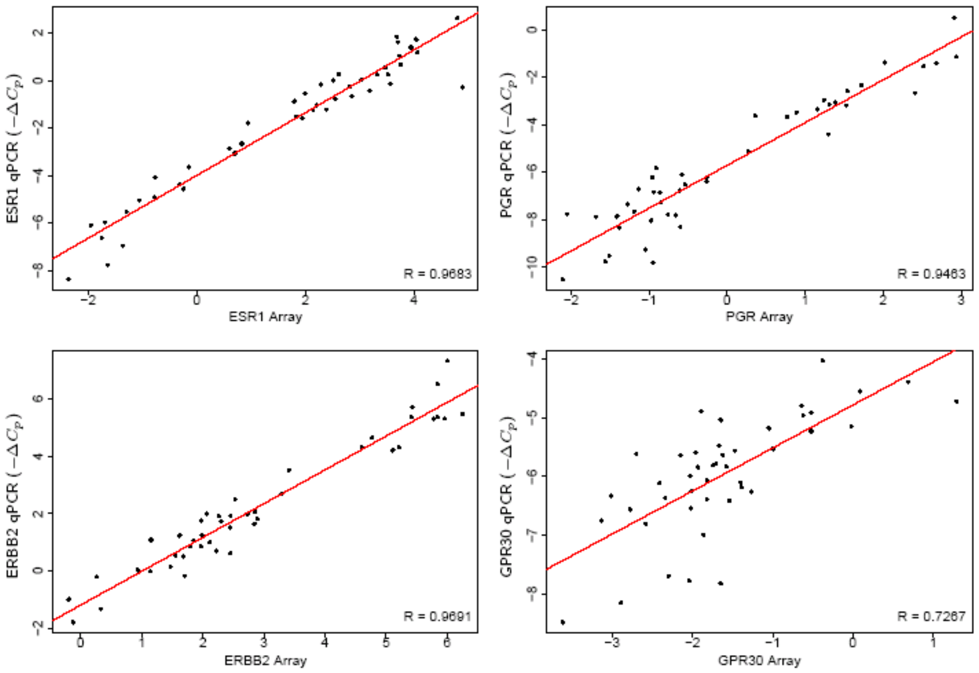

Figure S1. The quality control results of 48 clinical breast cancer gene expression arrays (48A) were derived by measuring the significance of linear associations between the relative quantitative mRNA levels of four chosen genes and their corresponding gene expression profiles in 48A. The values of correlation coefficient (R) for *ESR1*, *PGR*, *ERBB2* and *GPR30* are 0.9683, 0.9463, 0.9691 and 0.7267, respectively. The detailed information can be found in 'Methods' of the main article.

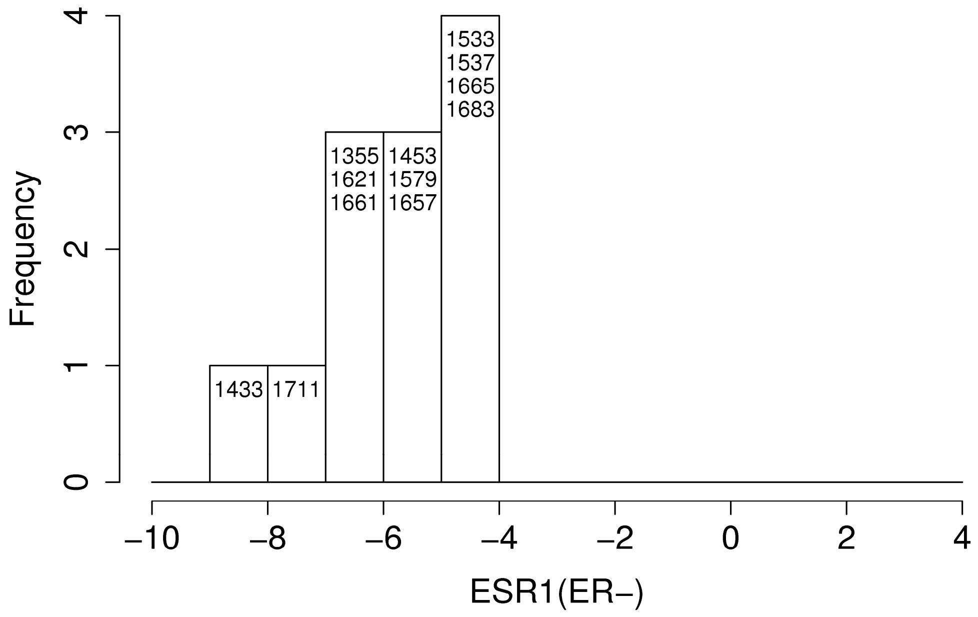

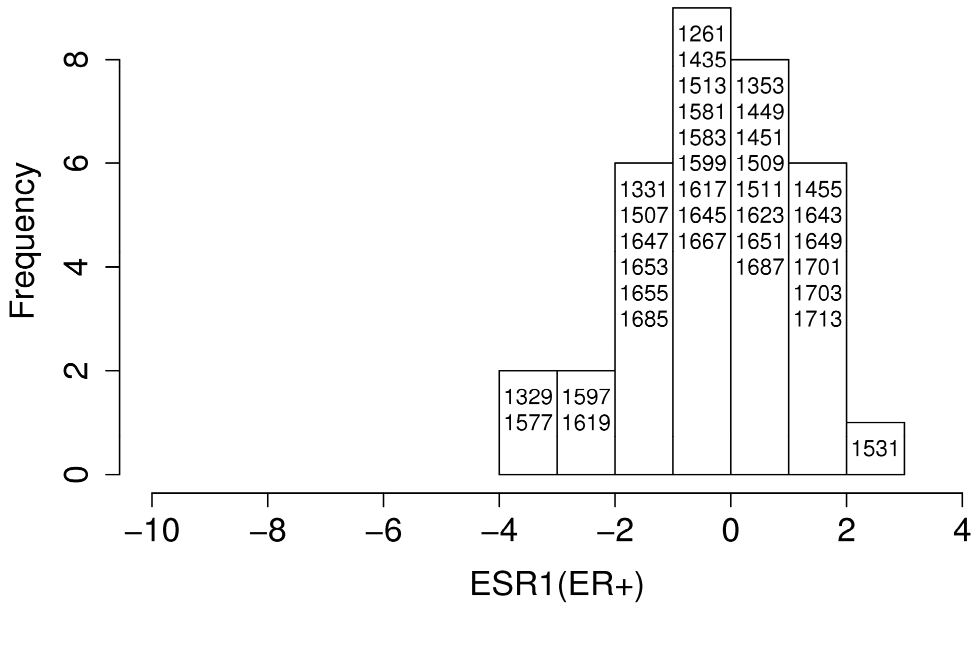

Figure S2. The histogram of *ESR1* mRNA levels for 48A based on the relative quantitative mRNA levels of each clinical sample detected by quantitative polymerase chain reactions (qPCR). The numbers in the histogram specify the ID of the arrays within each *ESR1* interval.

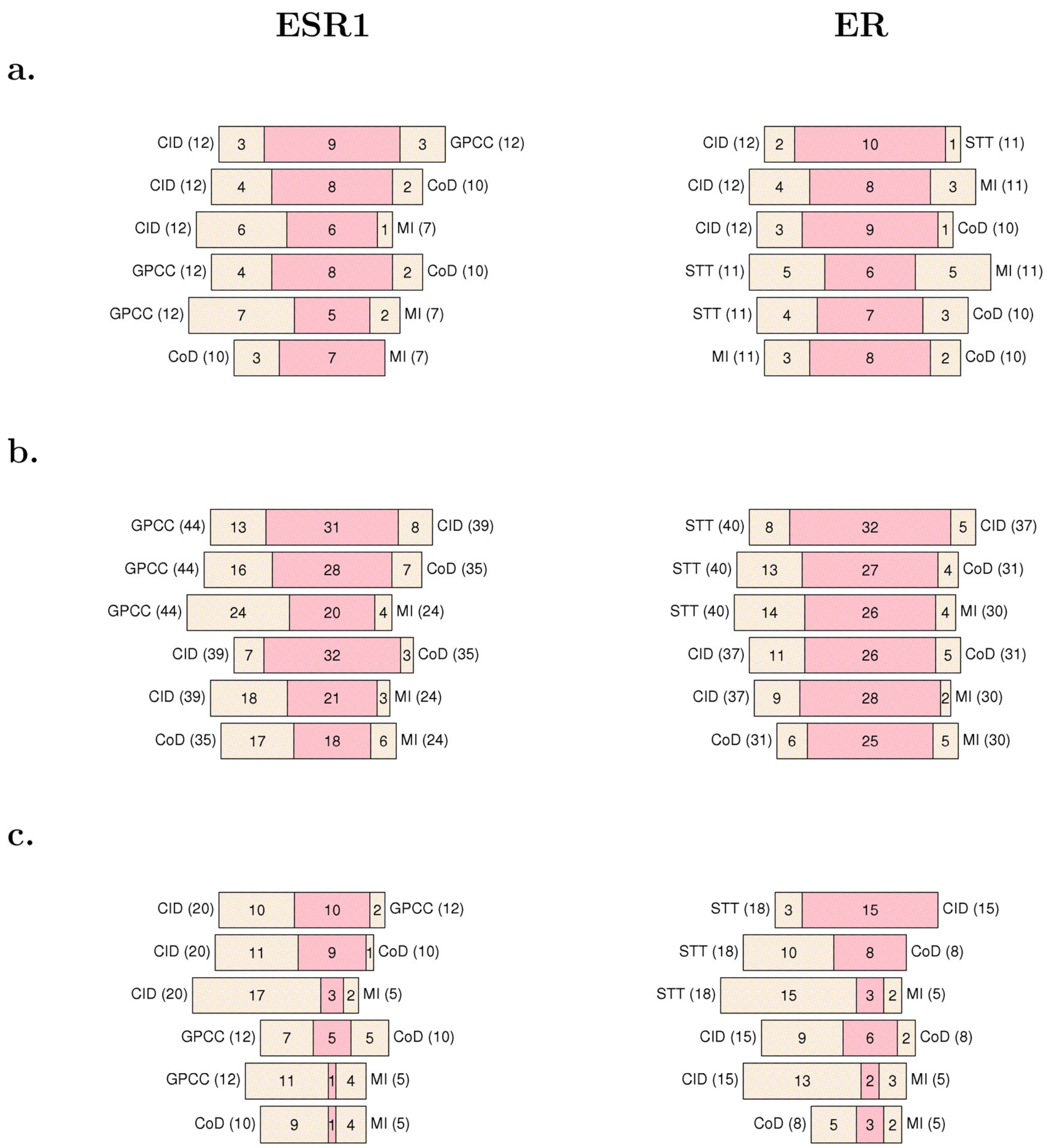

Figure S3. The comparison for numbers of significant genes claimed by different methods based on gene expression profiles in 48A. The pink bar located at the center represents the number of common genes found by the two methods compared: The light colored bars at both sides are unique genes extracted by methods described next to the bar. (a) results of *gene set I*; (b) results of *gene set II*; and (c) results of *gene set III*. The detailed information can be found in 'Methods' of the main article and Tables S2-S4 in this supplement.

Tables:

Table S1. The major features for five statistical methods used in this study (CID, Galton-Pearson's correlation coefficient, Student's *t*-test, coefficient of determination, and mutual information).

	**CID**	**GPCC**	**STT**	**CoD, MI**
**Assumption on distribution of variables**	No assumption	Normal distribution	Normal distribution	No assumption
**The descriptions of relationship among variables**	Both linear and non-linear relationships (Up- or down-stream association or two-way association)	Only linear relationship (Only with inversely or positively linear association)	The difference of means in two populations (Only with inversely or positively binary association)	Both linear and non-linear relationships (Up- or down-stream association or two-way association)
**Application**	Both continuous and discrete data	Only continuous data	Only discrete data	Only discrete data

Table S2. The summary of thirty three genes (*gene set I*) analyzed ($p \leq 0.05$ highlighted in blue) by eight statistical methods using 48A. The ranking score (floating points occur when there are ties) of each gene analyzed by these statistical methods is inside of parenthesis right after the *p* value, respectively.

* The average of ten associated probes is employed.

ESR1

Gene Symbol	Feature ID	CID-ESR1	GPCC	CoD-ESR1	MI-ESR1
ESR1	5561	0 (1)	0 (1)	0 (1)	0 (1)
BCL2	10911	0 (57)	0 (17)	0 (82)	0.01 (838)
PGR	11809	0 (220)	0 (440)	0 (151.5)	0.01 (821)
IGF1	9002	0 (486)	0.08 (5798)	0.01 (1144)	0.03 (1522)
LCN2	12033	0 (1191)	0 (1441)	0.02 (2093.5)	0.01 (652)
EGFR	2979	0 (1214)	0.14 (7443)	0 (800)	0.01 (961)
PTMA	16727	0.01 (1742)	0.01 (2611)	0.01 (1335)	0.29 (8307.5)
RAGE	13596	0.03 (3000.5)	0 (1228)	0.06 (3769)	0.54 (13202)
C3	12860	0.04 (3634.5)	0.03 (4042)	0.11 (5454)	0.57 (13709.5)
RARA	20979	0.04 (3682)	0 (2147)	0.04 (2971)	0.24 (7080)
BRCA1	*11046.4	0.05 (4015)	0.66 (15811)	0.28 (9716.5)	0.16 (5409.5)
TGFA	12974	0.05 (4039)	0 (795)	0.14 (6522)	0.28 (8050)
VEGF	1135	0.06 (4575)	0.04 (4585)	0.02 (2053)	0 (194.5)
IGFBP4	19106	0.06 (4611)	0 (947)	0.01 (1192)	0.22 (6766)
TFF1	2679	0.09 (5715)	0 (1751)	0.08 (4523)	0.36 (9645)
**No. of Significant Genes**		**12**	**12**	**10**	**7**

ER

Gene Symbol	Feature ID	CID-ER	STT	CoD-ER	MI-ER
LCN2	12033	0 (1098)	0.01 (1573)	0.49 (13692.5)	0 (225)
EGFR	2979	0 (1151.5)	0.18 (7443)	0.01 (1831)	0.03 (1997)
TGFA	12974	0 (1354)	0.02 (2534)	0.08 (4589.5)	0.16 (5741)
ESR1	5561	0 (2)	0 (1)	0 (2.5)	0 (2.5)
BCL2	10911	0 (30)	0 (38)	0 (39.5)	0 (79)
IGFBP4	19106	0 (328)	0 (294)	0 (187)	0 (371.5)
PGR	11809	0 (43.5)	0 (25)	0 (90)	0 (147)
TFF1	2679	0 (797.5)	0 (1042)	0 (637)	0.02 (1925)
VEGF	15367	0.02 (2397)	0.04 (3685)	0.02 (2159.5)	0.07 (3444)
GRIN1	17234	0.04 (3537.5)	0.02 (2422)	0.04 (3192)	0.18 (6212)
RAGE	13596	0.05 (3657)	0.04 (3690)	0.15 (6666.5)	0.41 (10933)
BRCA1	*11046.4	0.05 (3767.5)	0.2 (7746)	0.03 (2721.5)	0.01 (1177)
CTSD	3712	0.18 (7152)	0.85 (18117)	0.01 (1943.5)	0 (275)
LTF	11787	0.25 (8461)	0.05 (3959)	0.13 (6244.5)	0.2 (6657.5)
PTMA	4335	0.38 (10773)	0.1 (5453)	0.82 (18213.5)	0.05 (2900.5)
AGT	13948	0.4 (11198)	0.26 (8806)	0.64 (15701.5)	0.04 (2669)
**No. of Significant Genes**		**12**	**11**	**10**	**11**

Table S3. The summary of genes in *gene set II* analyzed ($p \leq 0.05$ highlighted in blue) by eight statistical methods using 48A. Only those genes claimed significant by at least one statistic are listed. The ranking score (floating points occur when there are ties) of each gene analyzed by these statistical methods is inside of parenthesis right after the p value, respectively.

ESR1

Gene Symbol	Feature ID	CID-ESR1	GPCC	CoD-ESR1	MI-ESR1
GREB1	5935	0 (12)	0 (13)	0 (19)	0 (9)
RERG	20434	0 (28)	0 (14)	0 (43)	0 (233)
MYB	5586	0 (35)	0 (37)	0 (6)	0 (240)
RBM24	14857	0 (46)	0 (182)	0 (383)	0 (185.5)
NPY1R	269	0 (73)	0 (55)	0 (82)	0 (77)
CA12	15121	0 (85)	0 (18)	0 (33.5)	0 (357.5)
STC2	12972	0 (97)	0 (77)	0 (140)	0.03 (1743)
SLC25A24	551	0 (162)	0 (810)	0 (169)	0.01 (658.5)
TPBG	16436	0 (208)	0 (108)	0 (161)	0 (21)
PGR	11809	0 (220)	0 (440)	0 (151.5)	0.01 (821)
RBBP8	19447	0 (351)	0 (1035)	0.01 (1695)	0.11 (4093)
RLN2	19701	0 (415)	0 (562)	0 (216)	0.11 (4138.5)
FLJ20366	20882	0 (428)	0 (617)	0 (326.5)	0 (183)
EFEMP1	22497	0 (1044)	0.82 (17962)	0.04 (2951)	0.3 (8471.5)
CCND1	2097	0 (1080)	0 (243)	0 (877)	0.07 (2760.5)
XBP1	10024	0 (1090)	0 (403)	0.01 (1111)	0 (423.5)
WFS1	9106	0 (1101)	0 (1485)	0 (341)	0.01 (946)
IL17RB	18755	0 (877.5)	0 (1581)	0 (559)	0.03 (1535)
MCM6	2749	0 (911)	0 (1428)	0 (618)	0.21 (6606)
CXCL12	20858	0 (975)	0.64 (15501)	0.03 (2551)	0.54 (13179)
IDH1	15099	0.01 (1536)	0 (299)	0 (826.5)	0.01 (615)
C14orf139	15004	0.01 (1615)	0.1 (6472)	0 (794)	0.04 (1919)
PAPSS2	16394	0.01 (1887)	0 (601)	0 (822.5)	0.06 (2626)
ANXA9	11320	0.02 (2213.5)	0 (1125)	0.05 (3374)	0 (467.5)
SYNE2	11324	0.02 (2500)	0.12 (6990)	0.03 (2717)	0.13 (4565)
TGM2	11119	0.02 (2746)	0.08 (5745)	0.04 (3189)	0.07 (2806)
UGCG	7423	0.02 (2825.5)	0 (1685)	0.04 (3174.5)	0.21 (6516.5)
ISG20	4186	0.03 (3041)	0.06 (4996)	0.1 (5202)	0.01 (861.5)
CELSR2	14077	0.03 (3048)	0.03 (3775)	0.36 (11190)	0.03 (1663.5)
PKIB	17511	0.03 (3075)	0.02 (3431)	0.12 (5923)	0.02 (1415.5)
RBP7	11680	0.03 (3102)	0.02 (3157)	0.13 (6093.5)	0.14 (4933.5)
SLC9A3R1	15223	0.03 (3296)	0.01 (2913)	0.04 (3110)	0.2 (6296)
WISP2	954	0.04 (3453)	0.26 (9776)	0.11 (5538.5)	0.71 (16140)
C22orf19	9354	0.04 (3466)	0 (1991)	0.16 (6960)	0.22 (6790.5)
ADAMTS19	1787	0.04 (3594)	0.15 (7522)	0.01 (1421)	0.11 (3918)
FKBP4	18433	0.04 (3768)	0 (773)	0.04 (3214)	0.05 (2333)
THRAP4	8979	0.04 (3878)	0.02 (3090)	0.07 (4391.5)	0.09 (3342)
KRT4	5398	0.05 (4070)	0.03 (3716)	0.04 (3026)	0.54 (13258.5)
SYTL4	14975	0.05 (4089)	0 (674)	0.04 (2995)	0.08 (3167.5)
IGFBP4	19106	0.06 (4611)	0 (947)	0.01 (1192)	0.22 (6766)
SLC29A1	5061	0.06 (4631)	0.01 (2951)	0.18 (7502)	0.09 (3385)
KIAA1324	19081	0.06 (4687.5)	0 (1126)	0.3 (10086)	0.06 (2478)
FER1L3	10227	0.06 (4698)	0 (1265)	0.14 (6478)	0.09 (3568)
SOX9	12855	0.07 (4789)	0.22 (9186)	0.06 (3875)	0.05 (2259)
RET	19784	0.08 (5236.5)	0.01 (2703)	0.09 (4830)	0.47 (11793)
BRP44L	2918	0.08 (5287)	0.03 (4161)	0.08 (4634)	0.33 (9128.5)
HIPK2	17324	0.08 (5338)	0.02 (3411)	0.24 (8812.5)	0.47 (11878)
SFXN2	1765	0.09 (5554)	0 (1649)	0.02 (2190.5)	0.23 (6943.5)
FLJ10847	8236	0.09 (5780)	0.81 (17788)	0.04 (3150)	0.18 (5798.5)
COL18A1	2296	0.09 (5816)	0.18 (8319)	0.32 (10474.5)	0.03 (1637)
LRIG1	13383	0.1 (5935)	0 (1028)	0.07 (4129.5)	0.01 (622)
RHOD	20938	0.19 (8679)	0.02 (3101)	0.29 (9763)	0.36 (9789)
FLJ30058	18550	0.22 (9209)	0.04 (4355)	0.41 (12134)	0.15 (4981.5)
CARD10	15654	0.28 (10569)	0.01 (2378)	0.27 (9384.5)	0.49 (12283)
UGT2B15	16804	0.31 (11229)	0.02 (3638)	0.19 (7668)	0.68 (15674)
**No. of Significant Genes**		**39**	**44**	**35**	**24**

Table S3. (*continued*)

ER

Gene Symbol	Feature ID	CID-ER	STT	CoD-ER	MI-ER
GREB1	5935	0 (1)	0 (1)	0 (2.5)	0 (2.5)
RERG	20434	0 (16)	0 (11)	0 (22)	0 (33.5)
CA12	18131	0 (17)	0 (14)	0 (13.5)	0 (10.5)
PGR	11809	0 (43.5)	0 (25)	0 (90)	0 (147)
CCND1	2097	0 (72)	0 (172)	0 (113)	0 (344.5)
RBM24	14857	0 (73.5)	0 (52)	0 (90)	0 (147)
NPY1R	269	0 (89.5)	0 (209)	0 (90)	0 (336)
IDH1	15099	0 (93)	0 (113)	0 (384.5)	0 (55.5)
IGFBP4	19106	0 (328)	0 (294)	0 (187)	0 (371.5)
STC2	12972	0 (431)	0 (124)	0 (1107)	0.02 (1728)
TPBG	16436	0 (467)	0 (505)	0.01 (2009.5)	0 (236)
MYB	5586	0 (495)	0 (963)	0 (187)	0 (491.5)
MCM6	2749	0 (749.5)	0 (709)	0.01 (1477.5)	0.01 (1308)
WFS1	9106	0 (809)	0 (1503)	0.01 (1595)	0 (661)
SOX9	12855	0 (886.5)	0 (497)	0 (543.5)	0 (440)
FER1L3	10227	0 (940)	0 (706)	0 (968.5)	0.02 (1478.5)
UGCG	7423	0 (941)	0 (1378)	0.01 (1657.5)	0.01 (1398.5)
ANXA9	11320	0 (971.5)	0 (1316)	0 (1188)	0 (491.5)
RLN2	19701	0 (1016)	0 (466)	0 (507.5)	0.04 (2636)
PAPSS2	16394	0 (1132)	0 (774)	0.01 (1703.5)	0 (114)
SYTL4	14975	0 (1304.5)	0 (1211)	0 (900.5)	0.03 (1943)
RET	19784	0 (1352)	0 (1403)	0.01 (1801.5)	0.07 (3364.5)
HIPK2	19590	0.01 (1580)	0.02 (2589)	0.25 (8936.5)	0.02 (1487)
SYNE2	11324	0.01 (2260)	0.03 (3013)	0.07 (4048)	0.31 (8987.5)
C6orf141	4353	0.02 (2320)	0.06 (4311)	0.03 (2705.5)	0.05 (2964.5)
FLJ20366	20882	0.02 (2395.5)	0.02 (2433)	0.03 (2928)	0.01 (1115)
XBP1	10024	0.02 (2399)	0.01 (2373)	0.09 (4882)	0.01 (1253)
RBP7	11680	0.02 (2423.5)	0.02 (2461)	0.01 (1409.5)	0.1 (4325)
WISP2	954	0.02 (2445.5)	0.01 (2219)	0.13 (6313)	0.1 (4228.5)
SFXN2	1765	0.02 (2453)	0.02 (2905)	0 (742.5)	0.05 (2821)
CARD10	15654	0.02 (2493)	0.01 (1713)	0.32 (10402)	0.04 (2466.5)
FKBP4	18433	0.02 (2535)	0.01 (2213)	0.23 (8519)	0.14 (5390)
TST	5953	0.03 (3061)	0.16 (6892)	0.92 (19343)	0.02 (1686)
CD33L3	14594	0.03 (3088)	0.18 (7395)	0.64 (15764)	0.15 (5466.5)
CXCL12	20858	0.03 (3264.5)	0.06 (4301)	0.22 (8125.5)	0.27 (8045.5)
SLC9A3R1	15223	0.04 (3330.5)	0.1 (5487)	0.14 (6393.5)	0.41 (10746)
BRP44L	2918	0.05 (3932)	0.05 (3923)	0.08 (4666.5)	0.1 (4358)
KCNK6	22031	0.06 (4123)	0.03 (3357)	0.02 (2334.5)	0.2 (6597)
RHOD	20938	0.07 (4435)	0.03 (3378)	0.03 (2843)	0.14 (5314)
ISG20	4186	0.08 (4671.5)	0.11 (5794)	0.03 (2899.5)	0.17 (6020)
SCARB1	22463	0.08 (4856.5)	0.04 (3859)	0.92 (19328)	0.41 (10859)
ABHD2	12761	0.09 (5058)	0.05 (3932)	0.4 (11959)	0.31 (8886)
FLJ30058	18550	0.1 (5370.5)	0.03 (3374)	0.23 (8519)	0.46 (11758)
TPD52L1	20138	0.1 (5452.5)	0.04 (3561)	0.6 (15109)	0.37 (10133)
GPSM1	5234	0.13 (5928)	0.95 (19501)	0.01 (1853.5)	0.03 (2258.5)
SLC25A24	551	0.16 (6701)	0.04 (3470)	0.19 (7487.5)	0.16 (5671)
SLC29A1	5061	0.17 (6914)	0.05 (3931)	0.95 (19611)	0.29 (8554)
ANKH	8237	0.42 (11486.5)	0.44 (11842)	0.57 (14750.5)	0.05 (2900.5)
PKIB	17511	0.43 (11546.5)	0.6 (14297)	0.05 (3640)	0.28 (8137)
**No. of Significant Genes**		**37**	**40**	**31**	**30**

Table S4. The summary of genes in *gene set III* analyzed ($p \leq 0.05$ highlighted in blue) by eight statistical methods using 48A. Only those genes claimed significant by at least one statistic are listed. The ranking score (floating points occur when there are ties) of each gene analyzed by these statistical methods is inside of parenthesis right after the p value, respectively.

* The average of ten associated probes is employed.

ESR1

Gene Symbol	Feature ID	CID-ESR1	GPCC	CoD-ESR1	MI-ESR1
ANLN	8789	0 (704)	0.01 (2441)	0.02 (1886)	0.2 (6239)
RRM2	11545	0 (1134)	0 (916)	0.01 (1694)	0.13 (4482)
DUT	16634	0.01 (1526)	0 (1986)	0.07 (4116.5)	0.22 (6723.5)
PLK4	17330	0.01 (1770)	0.31 (10625)	0.01 (1673)	0.22 (6847)
KIF11	18859	0.01 (1834)	0.14 (7478)	0.04 (2881)	0.28 (8100)
CHEK1	13123	0.01 (2023)	0.03 (4014)	0.14 (6483.5)	0.57 (13785.5)
CDC45L	12990	0.02 (2346.5)	0.01 (3048)	0.01 (1662)	0.02 (1061.5)
TTK	18156	0.02 (2376)	0.08 (5682)	0.03 (2548.5)	0.26 (7575.5)
HCAP-G	20741	0.02 (2644)	0.02 (3097)	0.1 (5148)	0.67 (15481.5)
FHL1	9568	0.02 (2792)	0.42 (12551)	0.22 (8347)	0.14 (4907.5)
PLAC1	3590	0.02 (2851)	0.01 (2153)	0.04 (2915)	0.2 (6185)
C10orf3	17003	0.03 (2946)	0.03 (4076)	0.14 (6436)	0.5 (12412)
MYBL2	10757	0.03 (3057.5)	0.01 (2992)	0.05 (3721)	0.79 (17278)
FSHPRH1	7641	0.03 (3385)	0.36 (11517)	0.05 (3572)	0.39 (10383.5)
PRC1	17954	0.04 (3516.5)	0.94 (19384)	0.15 (6832.5)	0.1 (3642.5)
KIF23	9169	0.04 (3621)	0.63 (15449)	0.06 (4023)	0.03 (1605)
KNTC2	11250	0.05 (3950)	0.02 (3195)	0.08 (4484.5)	0.74 (16548)
PCNA	13872	0.05 (4044)	0.42 (12489)	0.08 (4499)	0.53 (12971)
TOP2A	14222	0.05 (4075)	0.91 (19015)	0.18 (7402.5)	0.61 (14504)
CCNE2	11744	0.05 (4090)	0.68 (16015)	0.09 (5022)	0.03 (1650)
MELK	21145	0.06 (4335)	0.18 (8271)	0.07 (4413)	0.02 (1363)
BRIP1	14259	0.09 (5756)	0.82 (17898)	0.04 (3232)	0.14 (4878.5)
EXO1	* 12687.1	0.1 (5909)	0.03 (3678)	0.09 (4891.5)	0.57 (13726)
ASF1B	15126	0.24 (9720)	0.02 (3530)	0.29 (9773)	0.39 (10341)
OFCC1	5660	0.33 (11461)	0.28 (10225)	0.22 (8452)	0.03 (1648)
**No. of Significant Genes**		**20**	**12**	**10**	**5**

ER

Gene Symbol	Feature ID	CID-ER	STT	CoD-ER	MI-ER
ANLN	9296	0 (478.5)	0 (520)	0 (593)	0 (655)
RRM2	11545	0 (849)	0 (388)	0 (1081)	0.01 (953)
CDC45L	12990	0.01 (1503)	0.01 (1689)	0 (522)	0.06 (3097)
CHEK1	13123	0.01 (1754)	0.01 (2056)	0.11 (5299.5)	0.27 (7918)
EXO1	* 12687.1	0.01 (1758)	0.01 (1854)	0.13 (6313)	0.11 (4490.5)
FHL1	9568	0.01 (2150)	0.05 (3998)	0.38 (11678)	0.11 (4502)
C10orf3	17003	0.02 (2371.5)	0.01 (2134)	0.03 (2689)	0.11 (4592)
HCAP-G	20741	0.02 (2743.5)	0.01 (1725)	0.27 (9456)	0.25 (7593)
PLAC1	3590	0.03 (3025)	0.05 (4042)	0.08 (4445)	0.34 (9438.5)
KNTC2	11250	0.03 (3256)	0.01 (2049)	0.01 (1455.5)	0.09 (4049)
KIF11	18859	0.04 (3365)	0.02 (2555)	0.02 (2159.5)	0.21 (6804.5)
DUT	16634	0.04 (3435)	0.05 (4085)	0.34 (10935.5)	0.15 (5511)
FSHPRH1	7641	0.04 (3629.5)	0.03 (3310)	0.09 (4819.5)	0.27 (8060.5)
TTK	18156	0.05 (3720)	0.01 (2311)	0.07 (4214)	0.31 (9008.5)
MYBL2	10757	0.05 (3796)	0.05 (4047)	0.14 (6463)	0.31 (8950.5)
CDCA5	11468	0.07 (4466)	0.24 (8464)	0.5 (13781)	0.03 (2185.5)
MELK	21145	0.09 (5030.5)	0.02 (2676)	0.04 (3235)	0.05 (2881)
PLK4	17330	0.09 (5041.5)	0.04 (3865)	0.04 (3356.5)	0.15 (5553)
CDH2	18995	0.16 (6699)	0.04 (3520)	0.19 (7481.5)	0.19 (6466.5)
KIF23	9169	0.43 (11623)	0.21 (7938)	0.15 (6727)	0.02 (1762)
**No. of Significant Genes**		**15**	**18**	**8**	**5**

The supplementary text for Results and Discussions

A. The summary of thirty three genes identified by CID, GPCC, and STT using clinical breast cancer expression arrays for uncovering their regulatory association(s) with ESR1 and ER, respectively.

Enhanced ERα expression in the subgroup of malignant epithelium would induce more responsiveness to the proliferate effects of estrogens, but it remains to be determined whether estrogen continues to drive proliferation by the indirect mechanisms or whether an alternative pathway has arisen during malignant transformation *in vivo*.

By breaking down the layers of mystery for functional ERα activities in a tissue specific and cancer specific manner, the 48 breast cancer gene expression arrays provide the initial set of messages indicating functional ERα. Therefore, this message includes the ERE driven ERα target genes (e.g. *gene set I*) that can be used to classify the ER (+) subtypes and to lead the prediction of certain genotype as well as its corresponding phenotype based on ER (+) activated and/or ER (+) associated activities. CID measures the great variety of associations (briefly speaking, it is for both linear and non-linear as well as cause-result association between two variables. The variables can be either continuous or discontinuous). Therefore, once the gene of interest has been selected by CID on data from *in vivo* experiments and if it can also be supported by the *in vitro* established relationships between ERα and the gene of interest, then, it indicates the gene of interest having been identified to share the common association with ERα both *in vitro* and *in vivo*.

Below we summarize regulatory relationship(s) between ERα and the thirty three ERα target genes (Only data for CID-ESR1, CID-ER, GPCC-ESR1 and STT-ER are in Additional file 2. Others are not shown). We note that one unique property of CID in comparison with that of GPCC is measuring asymmetry. That means CID might yield different values to describe the association of Y given X and the association of X given Y, respectively. To further demonstrate the asymmetric property of CID, we switch the X and Y variables in the scatter plot and compute CID values from the opposite direction (see also 'Materials and Methods' in the main article). The CID values for other genes of interest are similarly derived. They are designated as CID-X, where X is presented as the symbol for the gene of interest.

a. Genes seen by CID only

IGF1 (AF#9002), a growth factor, is the target gene of ERα due to the ER binding site at its promoter region (Klinge 2001; O'Lone *et al,*2004). The predicted regulatory mechanism of *IGF1* mRNA expression was triggered in the presence of both DBP binding and ERα/ERE complex which was evident by a classification and regression tree (CART) model 2 (Jin *et al*, 2004). Therefore, *IGF1* was expected to be identified *in vivo* as a downstream factor of ERα. *IGF1* was found to be associated with ERα in a dual directional fashion by CID (CID-IGF1 for *ESR1*, $p = 0.016$ and CID-ESR1, $p = 0$). The finding in CID-IGF1 for *ESR1* can be supported biologically by the cross talking between *IGF1* mediated signaling and ERα mediated signaling which results in influencing ERα mRNA level *in vitro* (Stoica *et al,*2000), at least in part.

EGFR (AF#2979) is both an upstream effector influencing ERα expression and an ERα downstream activity including ERα driven *EGFR* expression seen by CID (Table S2 and Additional file 2). The cross-talking between growth factor signaling and estrogen signaling may be one of the major mechanisms which supports *EGFR* influencing ERα mRNA expression (Yang *et al,* 2004). Such *in vitro* evidence, in part, supports the finding by CID analyses (CID-EGFR for ESR1, $p = 0.034$; CID-EGFR for ER, $p = 0.042$).

BRCA1 (AF#11046.4) is an ERα direct transcriptional target gene (Klinge 2001; O'Lone *et al,* 2004) and statistically it can be identified by CID using *in vivo* data (Table S2). The proposed model for ligand status of the aromatic hydrocarbon receptor modulating transcriptional activation of *BRCA1* promoter by estrogen activated ERα was documented in breast cancer cell model- MCF-7 (Hockings *et al,* 2006). The tumor suppressor gene *BRCA1* encodes for a transcription factor that participates in regulation of cell proliferation and maintenance of genome integrity (Ma *et al,* 2007). It was found also an upstream effector in ERα regulatory mechanism by CID-BRCA1 for ER with $p = 0.002$. This may offer a brand new mechanism for the role of *BRCA1* in ER (+) breast cancer as well as in ER (+) normal breast. Previous studies indicated that BRCA1 physically interacts with ERα and inhibits its transcriptional activity including ERα mRNA expression (Fan *et al,* 1999; Fan *et al,* 2001; Fan *et al,* 2002; Kawai *et al,* 2002; Ma *et al,* 2005; Xu *et al,* 2005). Recent mechanistic study (Ma *et al,* 2007) indicated that c-Akt in growth factor signaling pathways conferred estrogen-independent ERα activation and rescued the *BRCA1* repression of estrogen-stimulated ERα activity including up-regulation of ERα mRNA level. Those regulatory mechanisms were identified, only in part, significantly by CID analyses in the cohort setting of 48A. CID suggests *BRCA1* mRNA expression to be suppressed by ERα.

b. ***Genes seen by either CID/STT or CID/GPCC***

VEGF (AF#1135) is the transcript variant 2 of *VEGF* (AF#15367). It is an ERα target gene product (CID-ER, $p = 0.034$). Its mRNA level was down-regulated in ER (+) population. However, this suppressive effect of ERα on *VEGF* (AF#1135) was not significant when its mRNA levels in two populations (i.e. ER (+) vs. ER (–)) were compared. The linear correlation coefficient showed an inverted association in both mRNA levels of ERα and of *VEGF* (AF#1135) in 48A (GPCC, $p = 0.045$).

PTMA (AF#16727) mRNA expression was found inversely associated with ERα mRNA expression (GPCC, $p = 0.01$). CID saw *PTMA* (AF#16727), transcript variant 2 of *PTMA* (AF#4335), to be the downstream factor of ERα *in vivo* (CID-ESR1, $p = 0.01$). That is consistent with the supporting evidence that *PTMA* is an ERα target gene. However, *PTMA* (AF#4335) mRNA expression was found lower in ER (+) group than in ER (–) group. In addition, it was not seen by CID as the causal factor of ERα. The research evidence (Covelo *et al,* 2006) suggested that *PTMA* may have the role in the structural modification of histones H3 and H4 which results in influencing chromatin activity. Therefore, *PTMA* influences ERα at transcriptome level that may be important to be discovered by CID when the N number of the cohort is increased.

GRIN1 (AF#17234) mRNA level was preferentially elevated in ER (+) of 48A (STT, $p = 0.016$). It was seen by CID-ER in 48A ($p = 0.042$). Therefore, CID suggested that *GRIN1* mRNA level may be up-regulated by ERα in 48A, at least in part.

C3 (AF#12860) has dual association patterns with ERα reflected in ER (+) breast cancer expression profiles seen by CID (CID-C3 for *ESR1*, $p = 0.036$; CID-ESR1, $p = 0.039$). It also showed the linear association with ERα and less mRNA expression level of *C3* in ER (+) population (GPCC, $p = 0.033$). The suppressive effect of ERα on *C3* mRNA expression, on contrary, an up-regulation of ERα mRNA may be due to *C3* directly or indirectly indicated by CID analyses. Such novel mechanism of mutual regulation between ERα and *C3* may provide more options for cancer prevention and treatments in ER (+) or ER (–) breast cancer subtypes.

CID saw the mRNA expression of *RARA* (AF#20979) as a downstream effect of ERα (CID-ESR1, $p = 0.04$). It is an ERα target gene which has been documented to have ERE at the promoter region of *RARA* gene (Klinge 2001; O'Lone *et al,* 2004). The inverted and linear association for the mRNA levels between ERα and *RARA* was re-identified by GPCC ($p = 0.005$). The repression of *RARA* mRNA expression by ERα was found *in silico* in 48A cohort setting.

c. ***Genes seen by student's t-test only***

LTF (AF#11787) was found low at mRNA level for ER (+) breast cancer subgroup. It was seen statistically significant by STT ($p = 0.048$). There was no significant association between *LTF* and ERα being found *in vivo* by GPCC and/or CID although it has ERE at its promoter region. This phenomenon deserves to be further analyzed both *in vitro* and *in vivo*.

d. ***Genes seen in three statistical methods***

LCN2 (AF#12033) is an ERE driven ERα target gene (Klinge 2001; O'Lone *et al,* 2004). It also has upstream association with ERα which was detected *in vivo* by CID (Table S2). This is a new regulatory mechanism which will be important in finding its roles in ERα biology, ERα cancer biology, and endocrine therapy. However, *LCN2* was down-regulated in ER (+) population which indicated the suppressive mechanisms for *LCN2* mRNA expression regulated by ERα. It was also suggested by CID (CID-LCN2 for *ESR1*, $p = 0.004$; CID-LCN2 for ER, $p = 0$) that the enhancing regulatory effect of *LCN2* on ERα mRNA expression. Finally, three methods all pointed that *LCN2* can be identified in ER (+) breast cancer indicating dual regulatory mechanism between ERα and *LCN2.* The research evidence for the causal effect of *LCN2* on ERα mRNA expression has not been documented yet.

PGR (AF#11809), a well known ERα target gene, was significantly identified by three statistical methods (Table S2). Interestingly, CID analyses showed a dual regulatory mechanism controlling both *PGR* and ERα mRNA expression. GPCC indicated the association of mRNA levels between *PGR* and *ESR1 in vivo* was positive. Biologically, PR-A (a *PGR* translated protein) is a transcription factor which is known to regulate a subset of PR-A target genes. The effect (s) of PR-A on up-regulation of ERα mRNA was observed by CID. It deserves further mechanistic study especially the research evidence of ours (Kuo *et al,* 2007) showed PR-A as the independent, good prognostic marker for breast cancer. The novel association of PR-A with ERα detected by CID, may uncover the set of gene activities associated with the good prognostic feature of PR-A in ER (+) group, in part.

BCL2 (AF#10911), an ERα target gene, was significantly identified by three statistical methods showing both causal (not shown) and resulting associations with ERα by CID analyses (Table S2 and Additional file 2). Biologically, *BCL2* is up-regulated by ERα, at least in part. However, *BCL2* up-regulates ERα mRNA

expression which mechanism is largely unknown. BCL2 is an antiapoptotic factor which is known to be associated with a set of gene activities contributing to part of the survival mechanisms (Burow *et al,* 2001). The new finding from CID indicates the dual regulatory mechanism which was not documented before.

ESR1 (AF#5561), an ERα target gene, was as the positive control for ER (+) population. The cut-off value for distinguishing ER (+) from ER (−) IDC was −4 (−ΔCp) (Figure S2). The specimens of our collection contained ~ 50% of tumor epithelial cells. *ESR1* (AF#20432), a transcript variant of ESR1 (AF#5561), was seen by four statistical analyses with significance ($p \leq 0.05$) like *ESR1* (AF#5561) does (see Additional file 2). It is expected that ERα can regulate its own mRNA expression as well as its transcript variant *ESR1* (AF#20432) in a dual regulatory manner but with different degrees of association (i.e. different ranking scores while the same *p*-value was observed). It was demonstrated by both *p*-values and ranking scores received for *ESR1* (AF#5561) and *ESR1* (AF#20432) by three methods applied (data not shown). To conclude our findings, the regulatory mechanism of both *ESR1* (AF#5561) and *ESR1* (AF#20432) in ER (+) breast cancer can be demonstrated both statistically and biologically.

VEGF (AF#15367) was found as the ERα target gene (Klinge 2001; O'Lone *et al,* 2004). Its mRNA level was significantly low in ER (+) breast cancer population (GPCC, $p = 0.05$; STT, $p = 0.041$). The association pattern between *VEGF* mRNA and ERα mRNA showed the linear relationship. CID also found *VEGF* (AF#15367) a resulting effect of ERα (CID-ER, $p = 0.017$) that can be translated as ERα down-regulating *VEGF* (AF#15367) at mRNA level, in part, in 48A. The true mechanism, both *in vitro* and *in vivo*, remains to be uncovered.

RAGE (AF#13596) was identified by all the statistical methods demonstrating that it has significant association with ERα (Additional file 2). The dual association seen by CID indicating a further investigation needed to find the causal effect of *RAGE* on ERα at transcriptional level. The mRNA levels between ERα and *RAGE* are positively, linearly associated (GPCC, $p = 0.001$). *RAGE* is preferentially expressed in ER (+) population (STT, $p = 0.04$).

TFF1 (AF#2679), also called *pS2*, is an early ERα regulated gene upon estrogen induction (Liu and Bagchi, 2004). It was also identified by three statistical methods *in silico* (Additional file 2). *TFF1* was also seen by CID as the causal effect for ERα (CID-TFF1 for ER, $p = 0.003$). Since there is no documented research evidence available yet, it would be a new direction in finding the role of *TFF1* which influences

the mRNA level of ERα in breast cancer setting.

TGFA (AF#12974) has ERE at its promoter region. It showed as the resulting factor of ERα *in silico* (CID-ER, p = 0.004; CID-ESR1, p = 0.048). It is important to know that *TGFA* was down-regulated in ER (+) breast cancer detected by GPCC (p = 0.003) (Additional file 2). It indicates *TGFA* to be down regulated by ERα observed *in vivo* (STT, p = 0.004).

IGFBP4 (AF#19106) was found significantly to have dual association with ERα by CID (CID-ER, p = 0; CID-IGFBP4 for ER, p = 0). The relationship between two gene expression patterns was identified *in silico*. *IGFBP4* has ERE at the promoter region. The CART model 2 also predicted the transcription of *IGFBP4* involving ERα and DBP (Jin *et al.* 2004). In addition, there is a novel association that *IGFBP4* is an upstream factor of ERα indicated by CID analysis. It should be worth a while to find the mechanism *in vitro* based on the finding from CID analyses in 48A. The mRNA levels of both ERα and *IGFBP4* are in a positive and linear association (GPCC, p = 0). *IGFBP4* mRNA level is preferentially higher in ER (+) population (STT, p = 0).

B. The summary of regulatory relationship(s) between ERα and the four cell cycle related estrogen responsive genes from the list of 302 genes which were derived from trajectory clustering (data not shown but partial results in Figure 4 of the main text).

CCNA2 (AF#8747.9), an example of estrogen secondary effect, reveals the feedback loop association with ERα *in vitro* (Jirstrőm *et al,* 2005). However, only downstream association with ERα by CID *in vivo* was observed for *CCNA2* in 48A (CID-ESR1, p = 0.018; CID-ER, p = 0.008; STT, p = 0.032). That means *CCNA2* mRNA expression being regulated by ERα. Another line of evidence from the prediction via a CART model 2, *CCNA2* can be transactivated in the presence of ERα binding incollaborating with DBP or GATA3 binding as the complex (Jin *et al,* 2004). The transcriptional expression of *CCNA2* is regulated by *E2F4* (Yochum *et al,* 2007). However, in the presence of estrogen, *E2F1* was documented to be the major transcriptional regulator and/or the co-regulator of genes mediating cell cycle (Stender *et al,* 2007). Our finding (result of trajectory clustering) and others (Stender *et al,* 2007) indicated that *CCNA2* can be up-regulated by *E2F1* in the presence of estrogen for breast cancer cell model-MCF-7. Surprisingly, the *E2F1* mRNA level was less in ER (+) population. Therefore, the result from CID analysis which *CCNA2* was less in ER (+) breast cancer population possibly, in part, due to *E2F1* was low in ER (+) group (see the

main text). In addition, it has negative regulatory association with *E2F2* (see Figure 4 of the main text).

CDC27 (AF#17078), a cell cycle gene, was not seen by all three methods with significance. Although its promoter can be activated by ERα in conjunction with other transcription factor (predicted by CART model 2), this is not significant in the finding of ours. Therefore, *CDC27* deserves to be further characterized in the cell model for the real mechanistic study as well as in clinical expression arrays, which have the larger *n* number ($n > 48$). Interestingly, *CDC27* was predicted not only to have negative regulatory association with *E2F2* but to have positive regulatory association with *E2F3* (see Figure 4). This evidence indicates *CDC27* to be a non-primary target of ERα.

CCNE2 (AF#11744) was found by CID as the downstream factor of *ESR1* demonstrated *in vivo* (CID-ESR1, $p = 0.05$ in Table S4). Biologically, the *in vitro* evidence (Moggs *et al,* 2005; Stender *et al,* 2007) also support, in part, our finding *in silico* that the transcriptional down regulation of *CCNE2* may be due to low levels of *E2F1*and/or *E2F4*. The further analysis of *E2F1* and *E2F4* in our cohort setting suggested the significant role of *E2F1* in suppressive regulation of *CCNE2* mRNA *in vivo* (GPCC, $p = 0.023976$, data not shown). Importantly, it also has the negative regulatory association with *E2F2* (see Figure 4 of the main text).

One of the transcriptional regulators of *PCNA* (AF#13872) is E2F4 (Yochum *et al,* 2007). The 5' promoter region of *PCNA* has an E2F1 binding site (Li *et al,* 2003). CID also significantly recognized *PCNA* having a downstream association with ERα (Table S4). *PCNA* is also a transcription factor to enhance ER/ERE complex formation (Schultz-Norton *et al,* 2007). This mechanism may include ERα transcriptional regulation itself. Such an upstream association with ERα was not recognized by CID in the ER (+) breast cancer clinical arrays. It should be highly interesting research topic which may be evident in a larger number of clinical expression arrays (n > 48). In Figure 4, it demonstrates a negative regulatory association between *E2F2* and *PCNA*.

Supplementary references for Results and Discussions.

Burow ME, Weldon CB, Tang Y, McLachlan JA, Beckman BS :**Oestrogen-mediated suppression of tumour necrosis factor alpha-induced apoptosis in MCF-7 cells: subversion of Bcl-2 by anti-oestrogens.** *J Steroid Biochem Mol Biol*

2001,**78**: 409-418.

Covelo G, Sarandeses CS, Diaz-Julien C, Freire M: **Prothymosin alpha interacts with free core histones in the nucleus of dividing cells.** *J Biochem* 2006, **140**: 627-637.

Fan S., Wang J, Yuan R, Ma Y, Meng Q, Erdos MR, Pestell RG, Yuan F, Auborn KJ, Goldberg I.D, Rosen EM: **BRCA1 inhibition of estrogen receptor signaling in transfected cells.** *Science* 1999, **284** : 1354-1356.

Fan S, Ma YX, Wang C, Yuan RQ, Meng Q, Wang JA, Erdos M, Goldberg ID, Webb P, Kushner PJ, Pestell RG, Rosen EM: **Role of direct interaction in BRCA1 inhibition of estrogen receptor activity.** *Oncogene* 2001, **20**:77–87.

Fan S, Ma YX, Wang C, Yuan RQ, Meng Q, Wang JA, Erdos M, Goldberg ID, Webb P, Kushner PJ, Pestell RG, Rosen EM: **p300 Modulates the BRCA1 inhibition of estrogen receptor activity.** *Cancer Res* 2002, **62**:141–151.

Hockings JK, Thorne PA, Kemp MQ, Morgan SS, Selmin O, Romagnolo DF: **The ligand status of the aromatic hydrocarbon receptor modulates transcriptional activation of BRCA-1 promoter by estrogen.** *Cancer Res* 2006, **66**: 2224-2232.

Jin VX., Leu YW, Liyanarachchi S, Sun H, Fan M, Nephew KP, Huang THM, Davuluri RV: **Identifying estrogen receptor alpha target genes using Integrated computational genomics and chromatin immunoprecipitation microarray.** *Nucleic Acids Res* 2004, **32**: 6627-6635.

Jirstrőm K, Stendah M, Ryde´n L, Kronblad A, Bendah PO, Sta°l O, Landberg G. : **Adverse effect of adjuvant tamoxifen in premenopausal breast cancer with cyclin D1 gene amplification.** *Cancer Res* 2005, **65**: 8009-8016.

Kawai H, Li H, Chun P, Avraham S, Avraham HK: **Direct interaction between BRCA1 and the estrogen receptor regulates vascular endothelial growth factor (VEGF) transcription and secretion in breast cancer cells.** *Oncogene* 2002, **21**: 7730-7739.

Klinge CM: **Estrogen receptor interaction with estrogen response elements**. *Nucleic Acids Res* 2001, **29**: 2905-2919.

Liu X, Bagchi MK: **Recruitment of distinct chromatin-modifying complexes by tamoxifen-complexed estrogen receptor at natural target gene promoters in vivo.** *J Biol Chem* 2004, **279**: 15050-15058

Li YY, Wang L, Lu CD: **An E2F site in the 5 '-promoter region contributes to serum-dependent up-regulation of the human proliferating cell nuclear antigen gene.** *FEBS Lett* 2003, **544**: 112-118.

Ma Y, Hu C, Riegel AT, Fan S, Rosen EM: **Growth factor signaling pathways modulate BRCA1 repression of estrogen receptor-alpha activity.** *Mol Endocrinol* 2007, **21**: 1905-1923.

Ma YX, Tomita Y, Fan S, Wu K, Tong Y, Zhao Z, Song LN, Goldberg ID, Rosen EM : **Structural determinants of the BRCA1: estrogen receptor interaction.** *Oncogene* 2005, **24**: 1831-1846.

Moggs JG, Murphy TC, Lim FL, Moore DJ, Stuckey R, Antrobus K, Kimber I Orphanides G.: **Anti-proliferative effect of estrogen in breast cancer cells that re-express ER alpha is mediated by aberrant regulation of cell cycle genes.** *J Mol Endocrinol* 2005, **34**:535-551.

O'Lone R, Frith MC, Karlsson EK, Hansen U: **Genomic targets of nuclear estrogen receptors.** *Mol Endocrinol* 2004, **18**: 1859-1875.

Schultz-Norton JR, Gabisi VA, Ziegler YS, McLeod IX, Yates JR and Nardulli AM: **Interaction of estrogen receptor alpha with proliferating cell nuclear antigen.** *Nucleic Acids Res* 2007, **35**: 5028-5038.

Stender JD, Frasor J, Komm B, Chang KCN, Kraus WL, Katzenellenbogen BS: **Estrogen-regulated gene networks in human breast cancer cells: Involvement of E2F1 in the regulation of cell proliferation.** *Mol Endocrinol* 2007, **21**: 2112-2123.

Stoica A, Saceda M, Fakhro A, Joyner M, Martin MB: **Role of insulin-like Growth factor-1 in regulating estrogen receptor-alpha gene expression.** *J Cell Biochem* 2000, **76**: 605-614.

Xu J, Fan S, Rosen EM: **Regulation of the estrogen-inducible gene expression profile by the breast cancer susceptibility gene BRCA1.** *Endocrinology* 2005, **146**: 2031-2047.

Yang Z, Barnes CJ, Kumar R: **Human epidermal growth factor receptor 2 status modulates subcellular localization of and interaction with estrogen receptor alpha in breast cancer cells**. *Clin Cancer Res* 2004, **10**: 3621-3628.

Yochum GS, Cleland R, McWeeney S, Goodman RH: **An antisense transcript induced by Wnt/beta-catenin signaling decreases E2F4.** *J Biol Chem* 2007, **282**: 871-878.
