# Supplementary material for: Statistical identification of gene association by CID in application of constructing ER regulatory network
Source: BMC Bioinformatics. 2009 Mar 17;10:85. doi: 10.1186/1471-2105-10-85 (PMC2679734; doi:10.1186/1471-2105-10-85)
Supplement: Additional file 2 — Plot analyses for gene set I. This file contains graphical illustration of statistical analyses for those genes appeared in gene set I. Four plots for each gene represent four tests (CID-ESR1, CID-ER, GPCC, and STT), respectively. [file 1471-2105-10-85-S2.pdf]

This file contains graphical illustration of analyses in 48A for those genes appeared in *gene set I*. Four plots for each gene represent four tests (CID-ESR1, CID-ER, GPCC, and STT), respectively. The number in the parentheses after the gene names are their feature numbers in Agilent Human 1A (version 2) oligonucleotide microarray.

ESR1

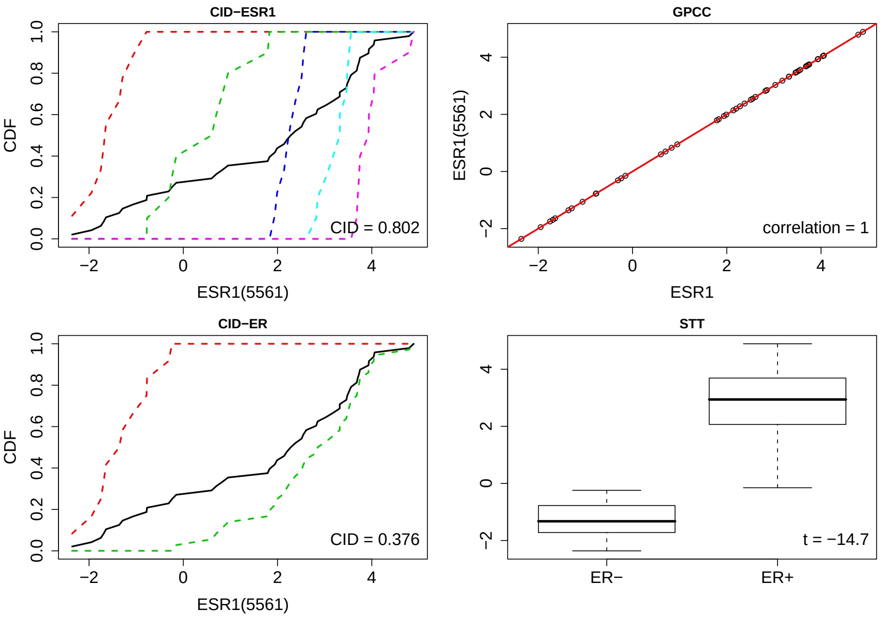

BCL2

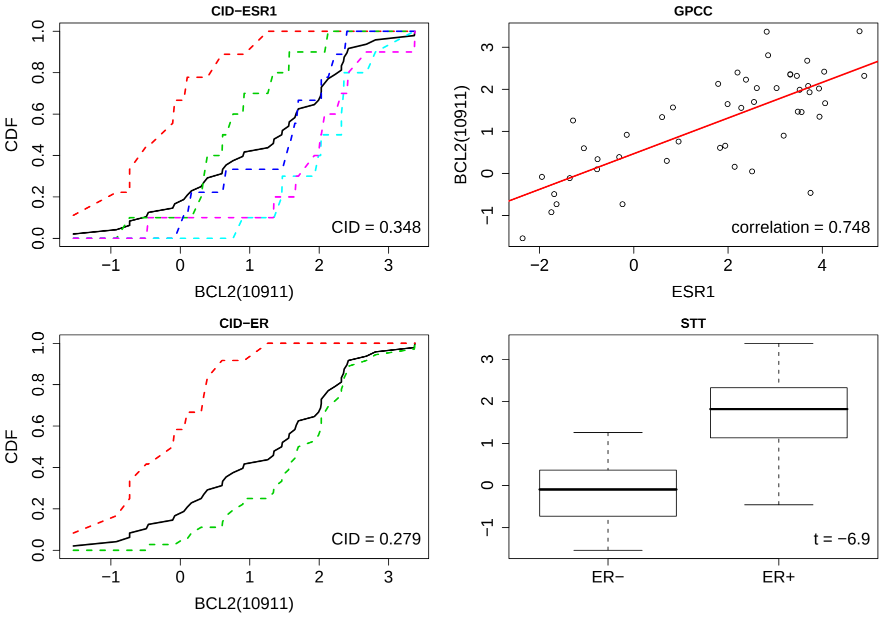

PGR

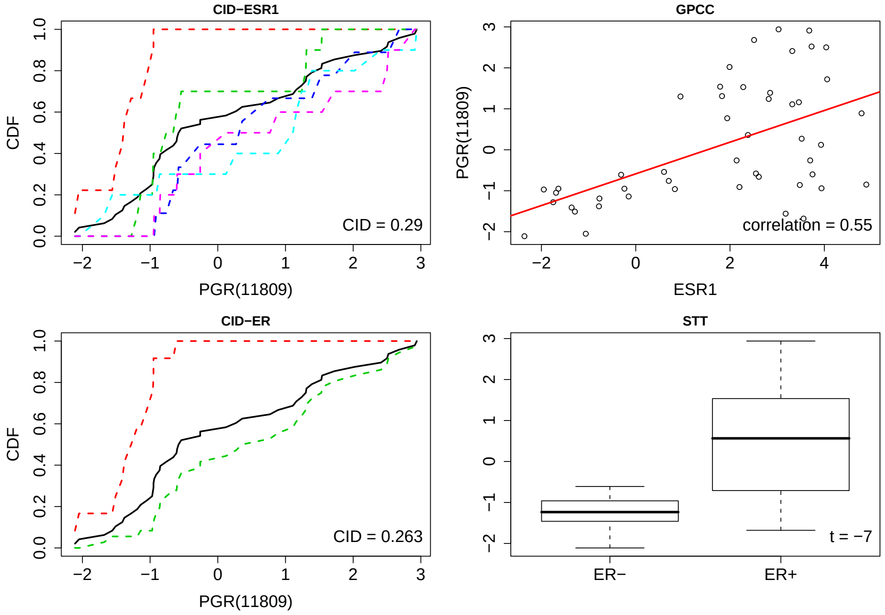

IGF1

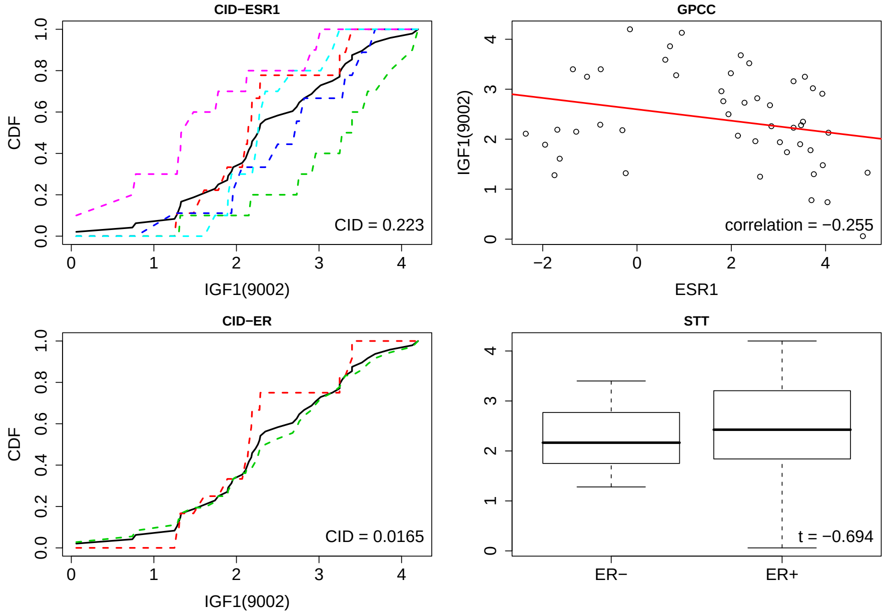

LCN2

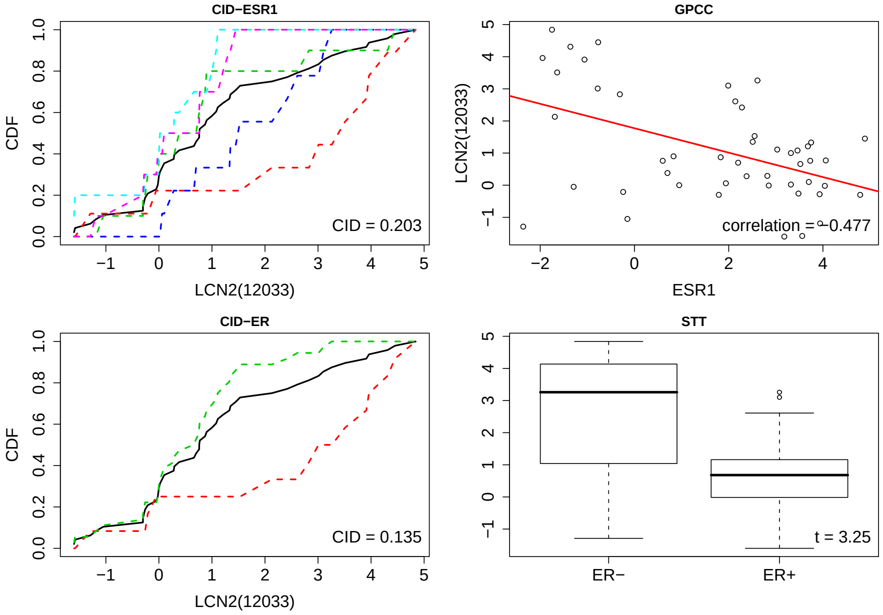

EGFR

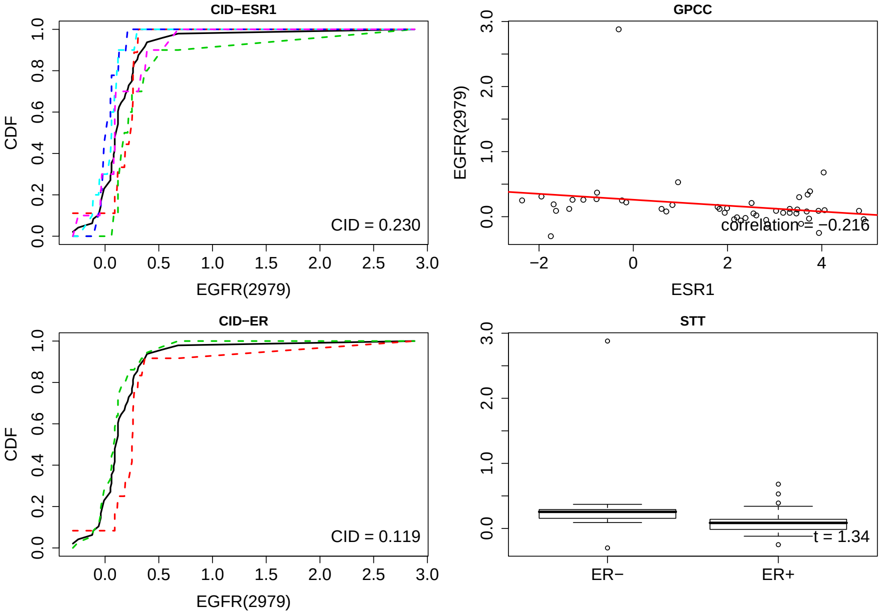

PTMA

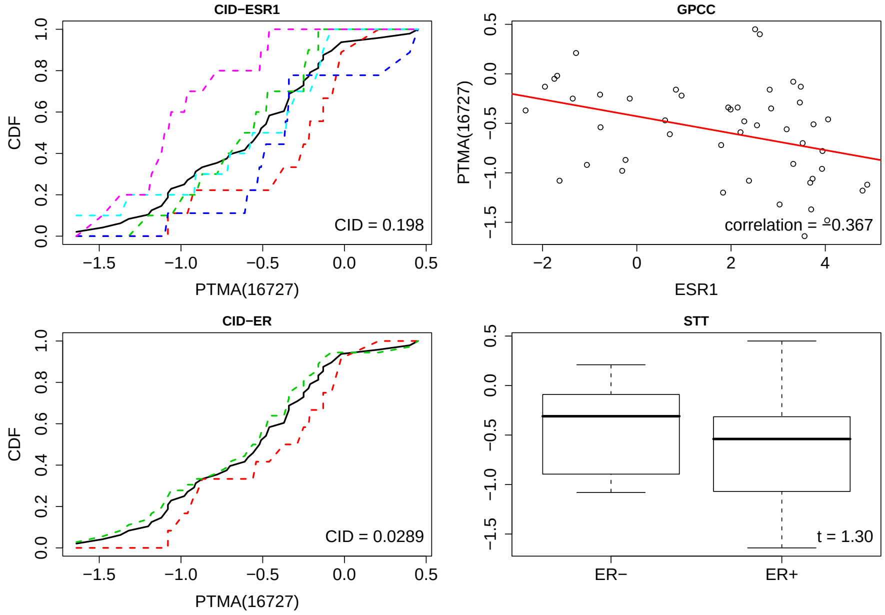

RAGE

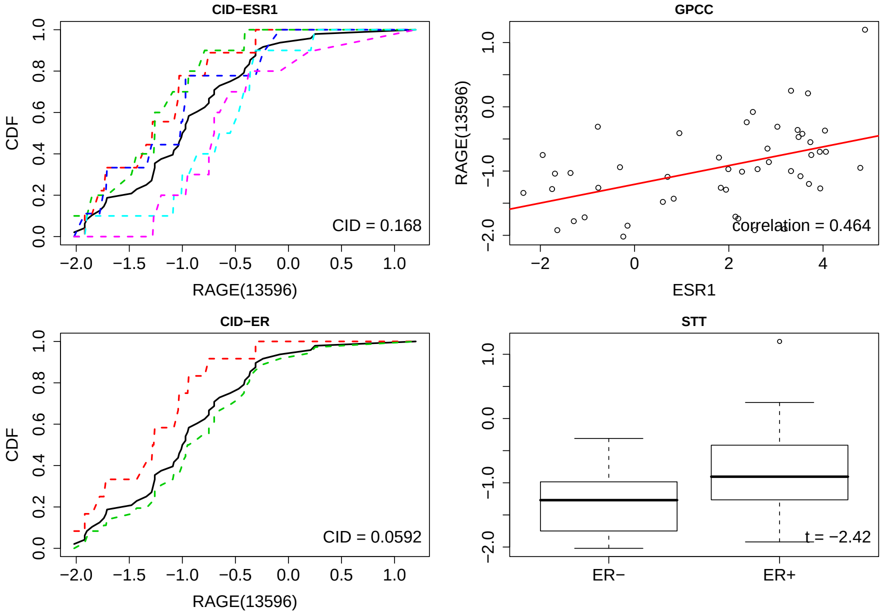

C3

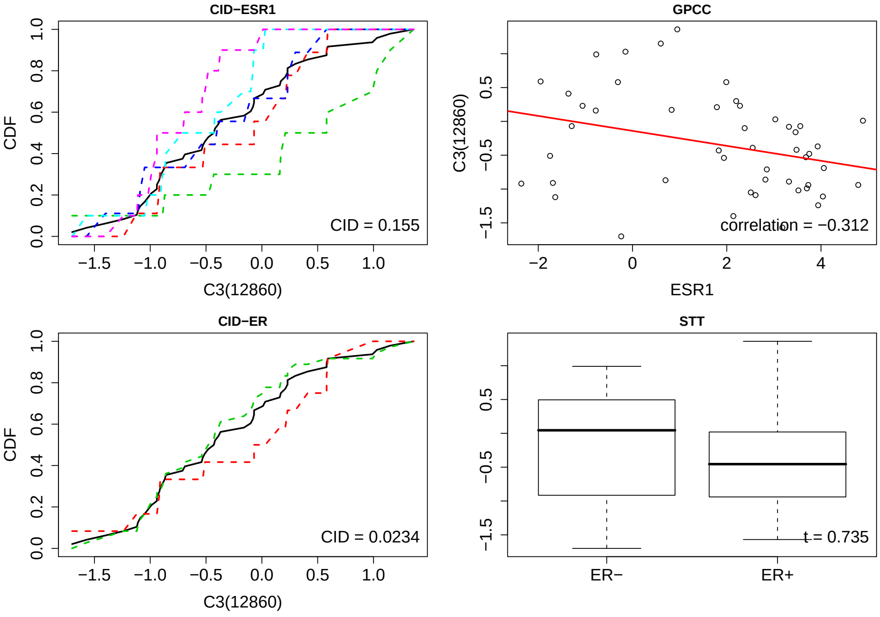

RARA

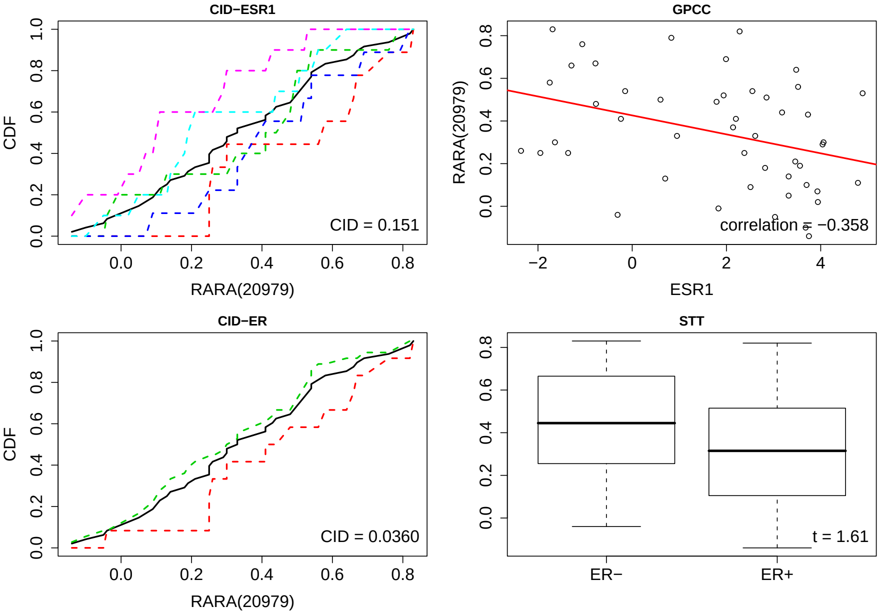

BRCA1

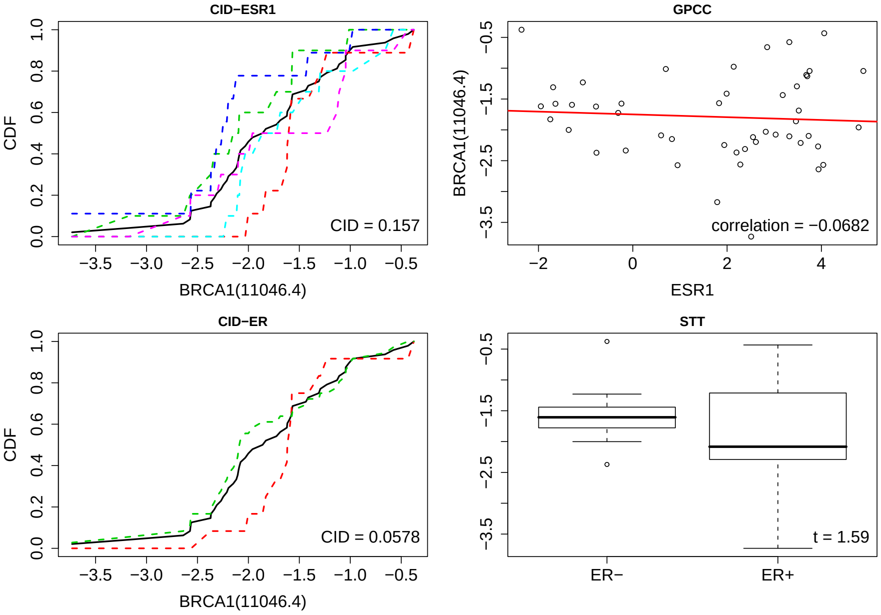

TGFA

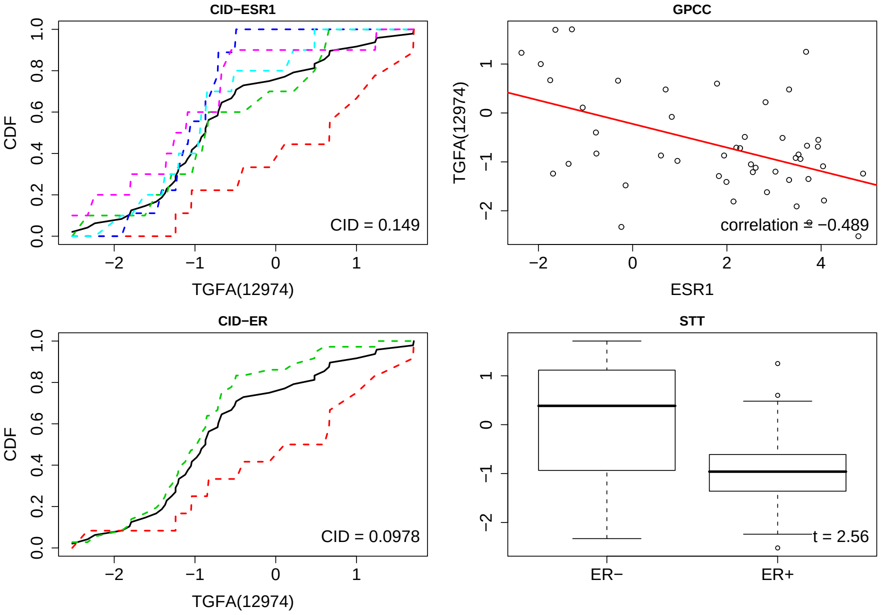

VEGF

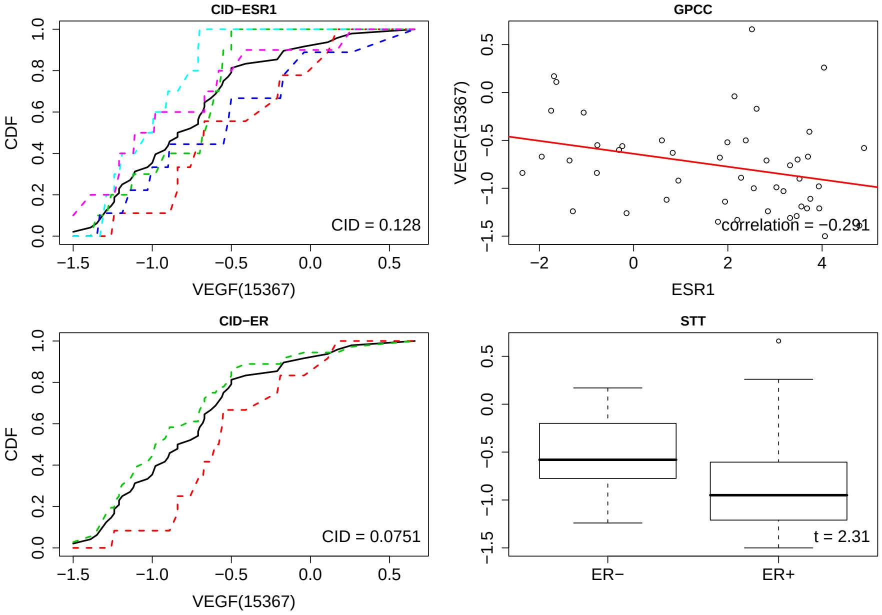

IGFBP4

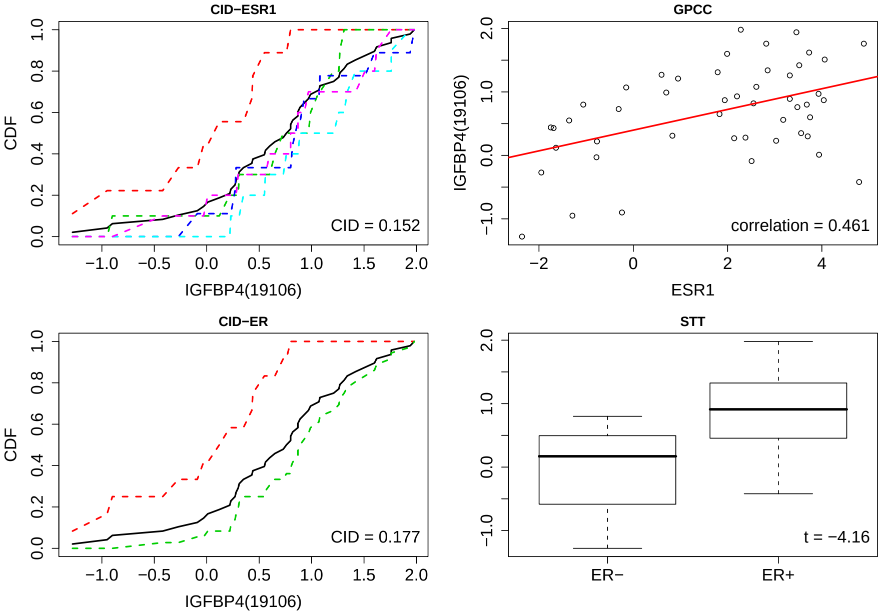

HSPB1

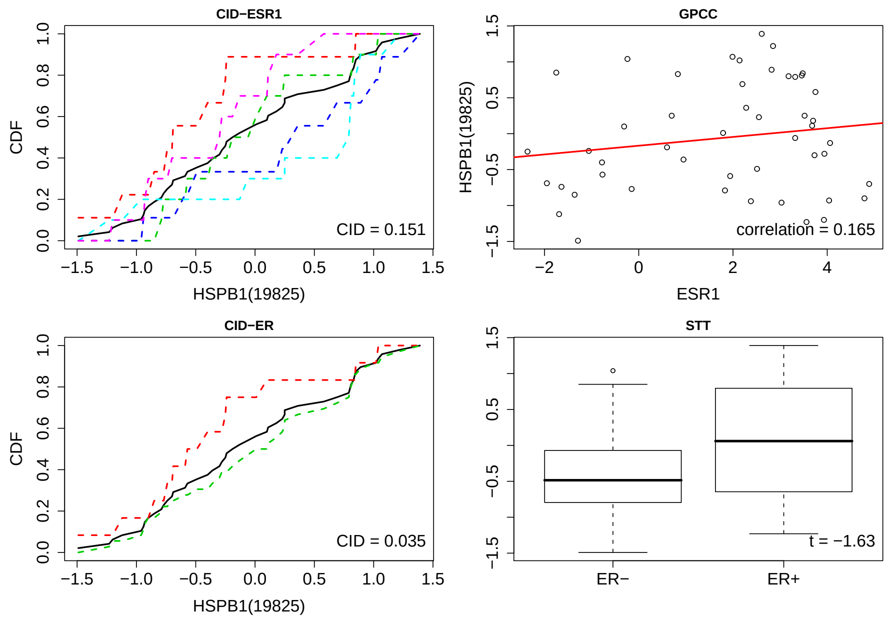

TFF1

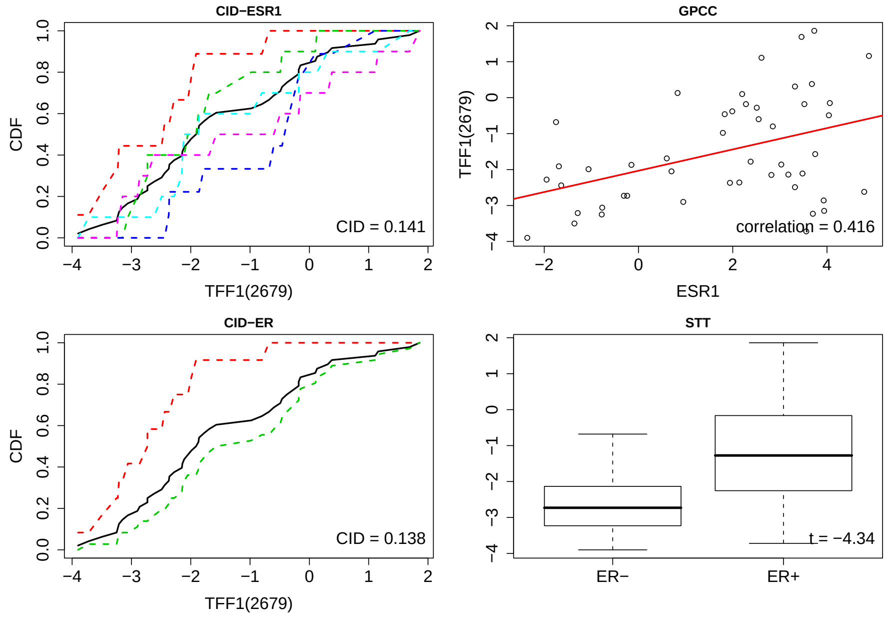

GRIN1

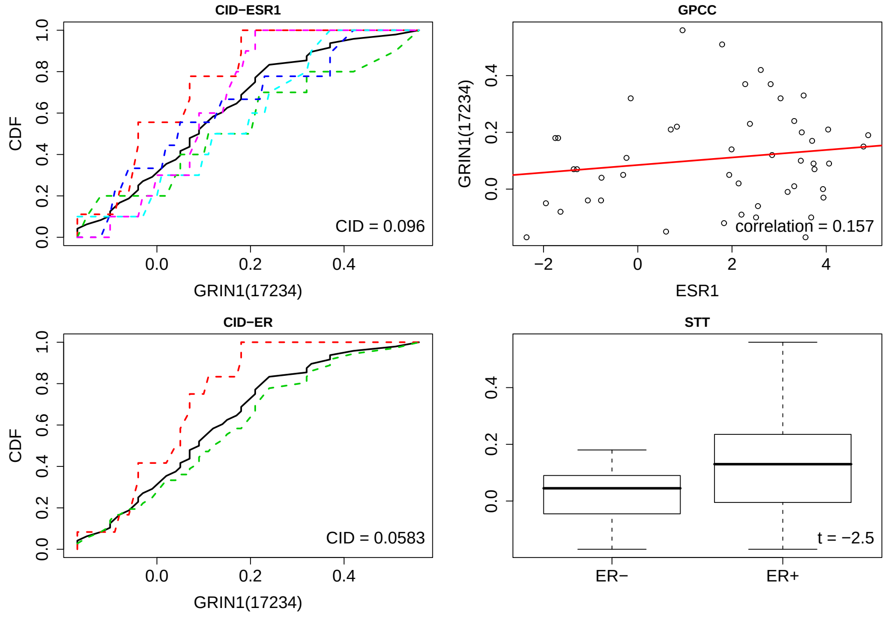

COX7A2L

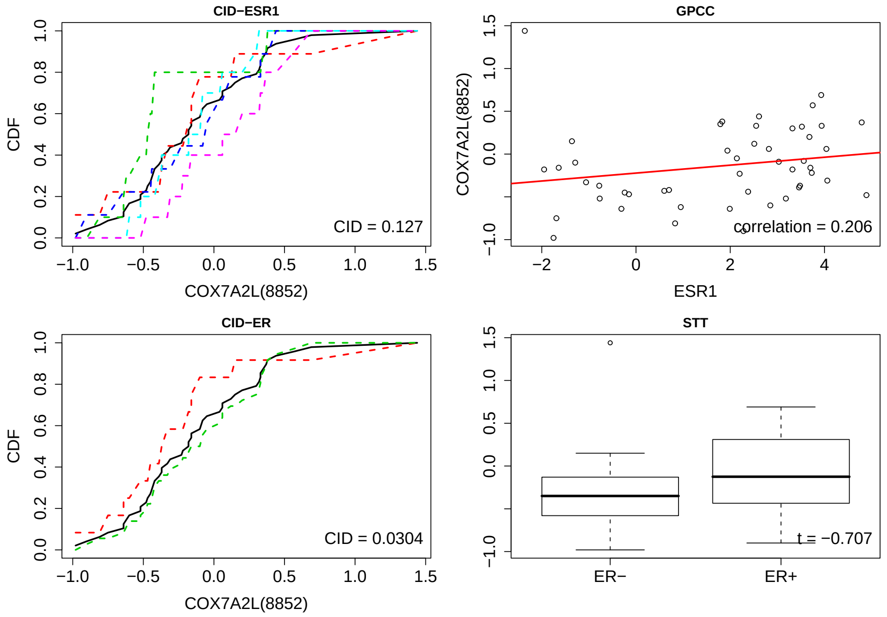

TYMS

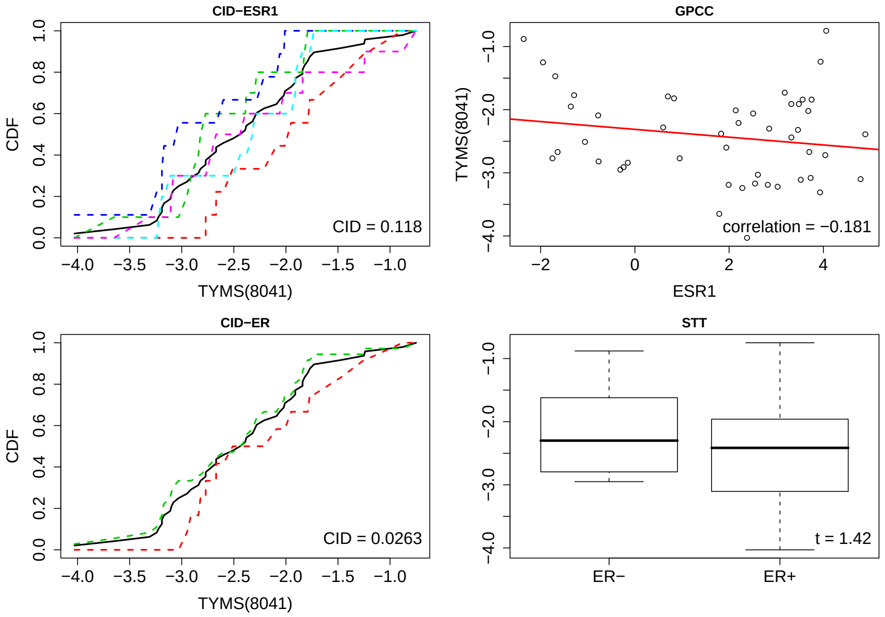

ADARB1

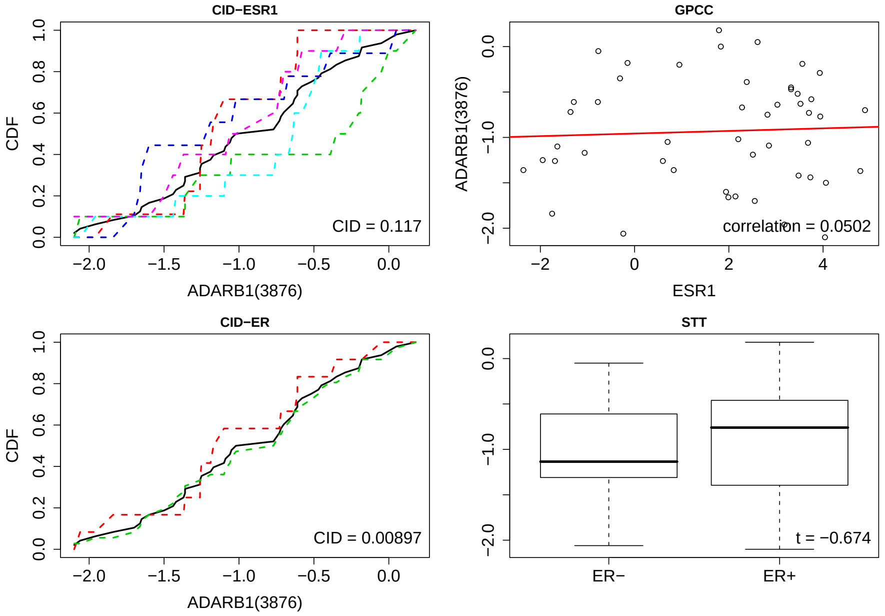

FOS

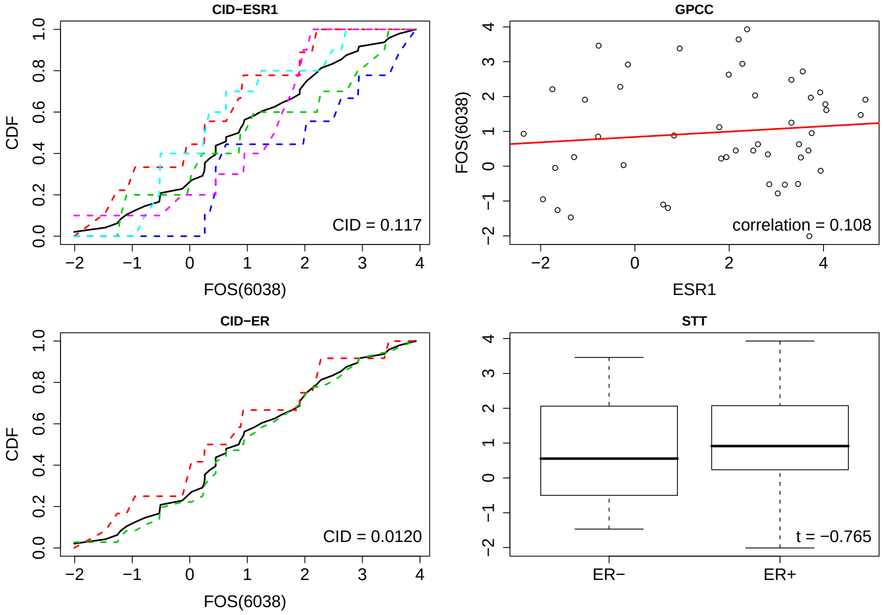

OXT

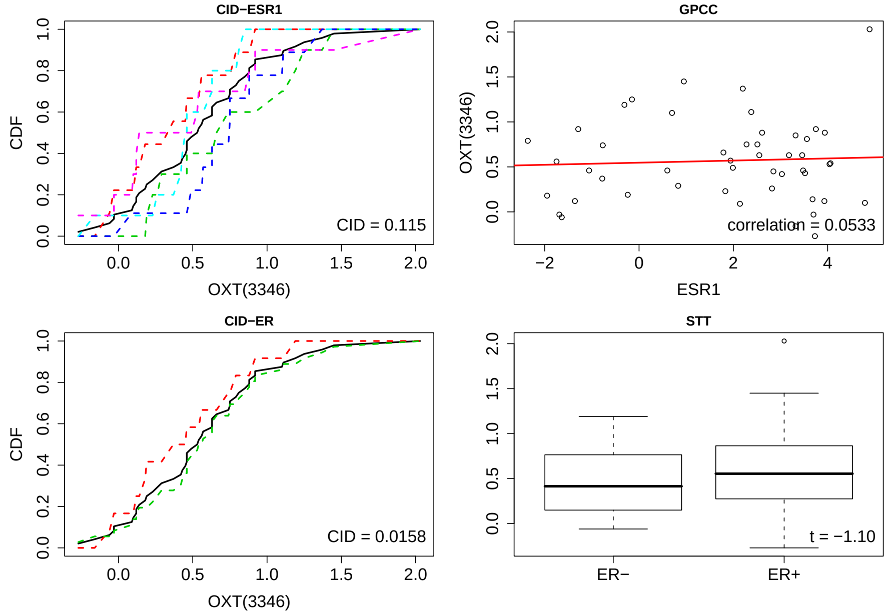

KRT19

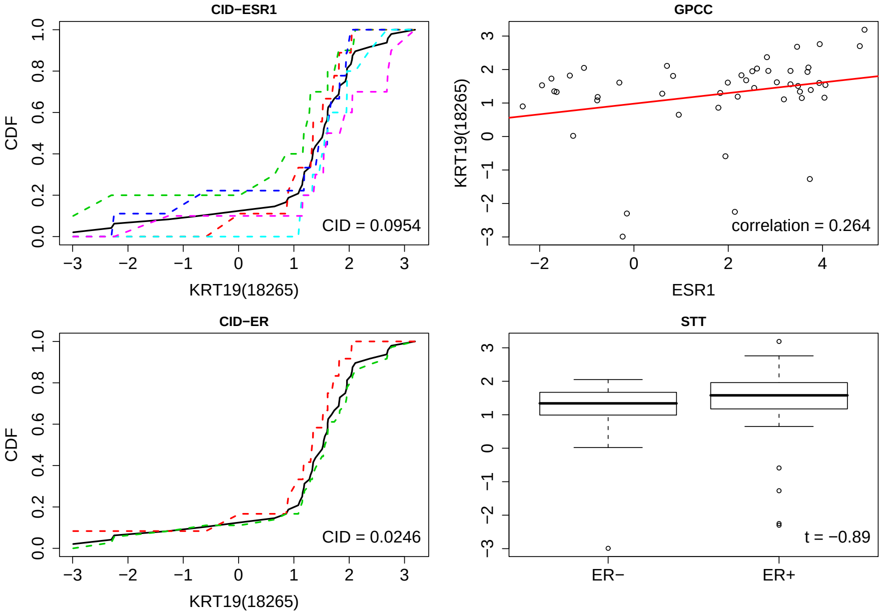

TERT

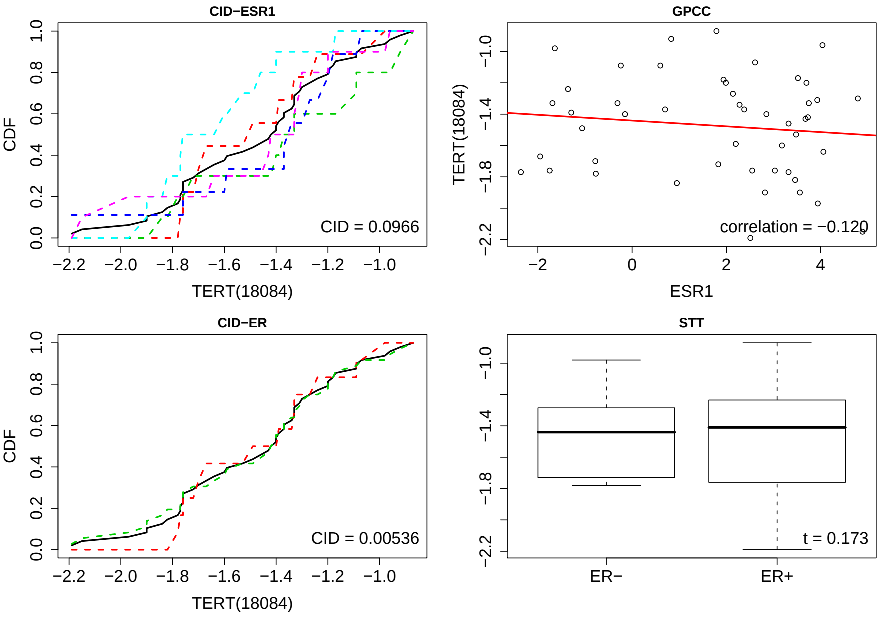
CID−ESR1
CDF
TERT(18084)
CID = 0.0966
GPCC
TERT(18084)
ESR1
correlation = −0.120
CID−ER
CDF
TERT(18084)
CID = 0.00536
STT
ER−
ER+
t = 0.173

MYC

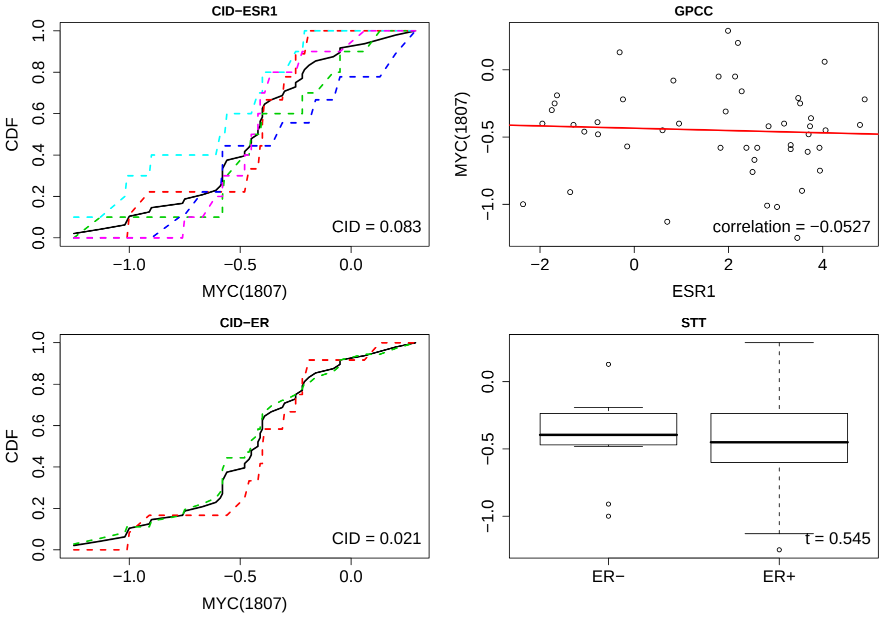
CID−ESR1
CDF
MYC(1807)
CID = 0.083
GPCC
MYC(1807)
ESR1
correlation = −0.0527
CID−ER
CDF
MYC(1807)
CID = 0.021
STT
ER−
ER+
t = 0.545

LTF

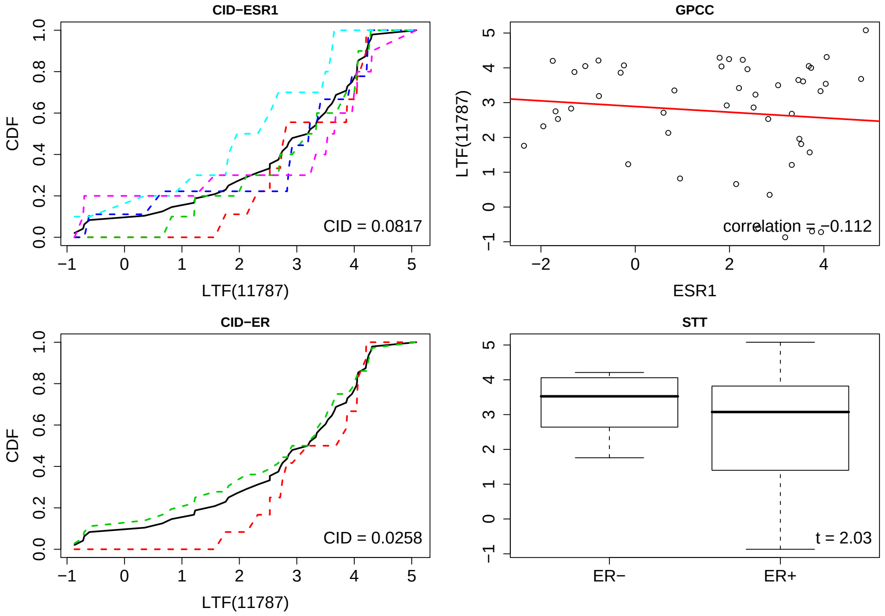
CID−ESR1
CDF
LTF(11787)
CID = 0.0817
GPCC
LTF(11787)
ESR1
correlation = −0.112
CID−ER
CDF
LTF(11787)
CID = 0.0258
STT
ER−
ER+
t = 2.03

CTSD

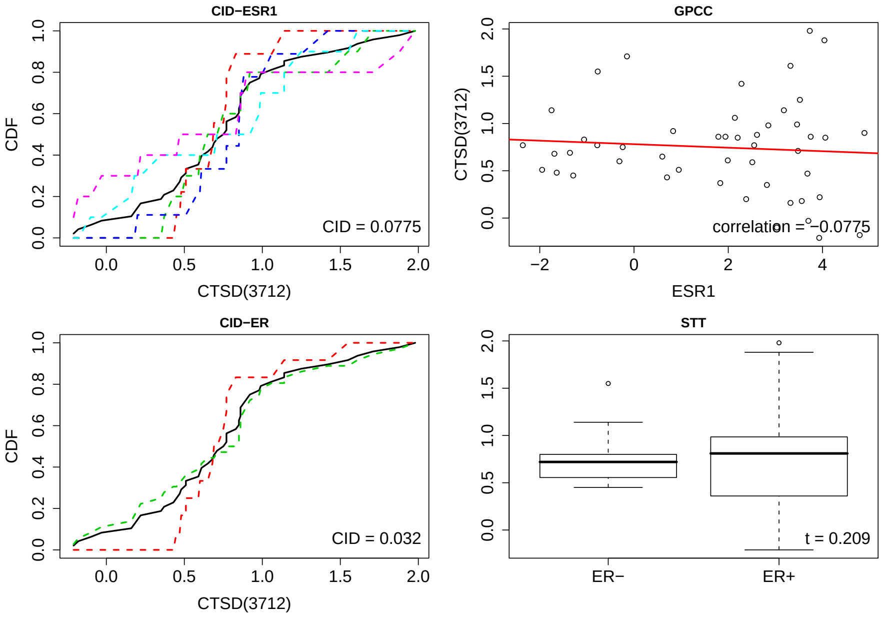

OVGP1

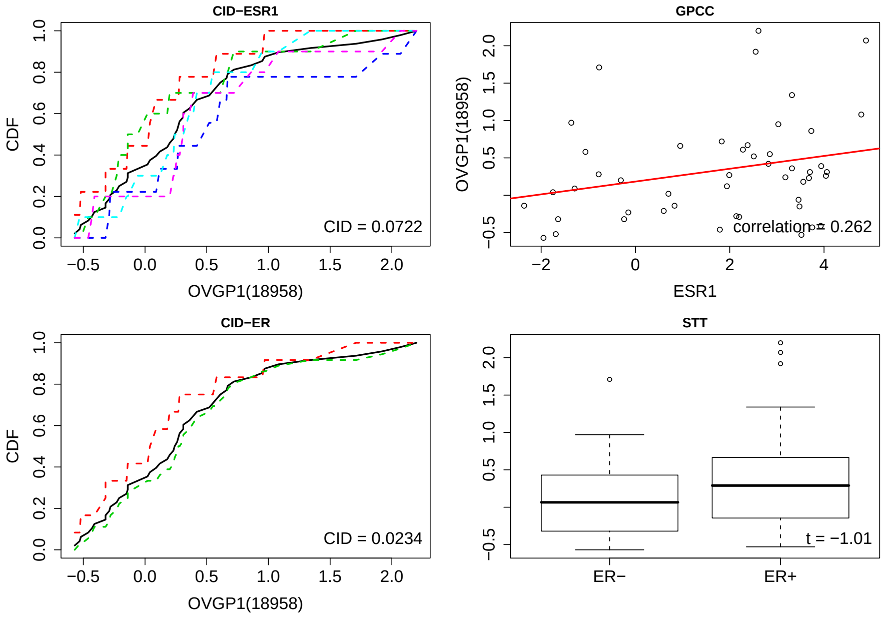

EBAG9

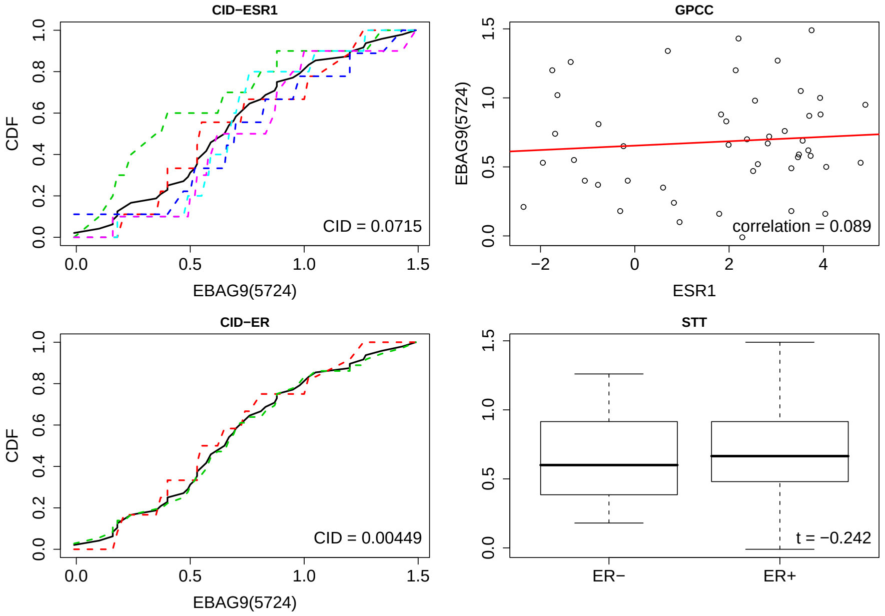

LDLR

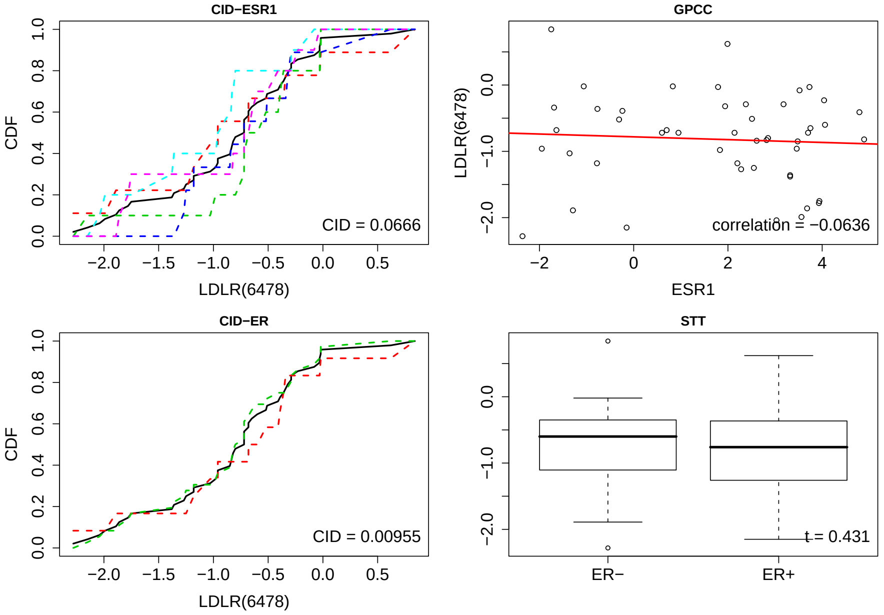
CID−ESR1
CDF
LDLR(6478)
CID = 0.0666
GPCC
LDLR(6478)
ESR1
correlation = −0.0636
CID−ER
CDF
LDLR(6478)
CID = 0.00955
STT
ER−
ER+
t = 0.431

F12

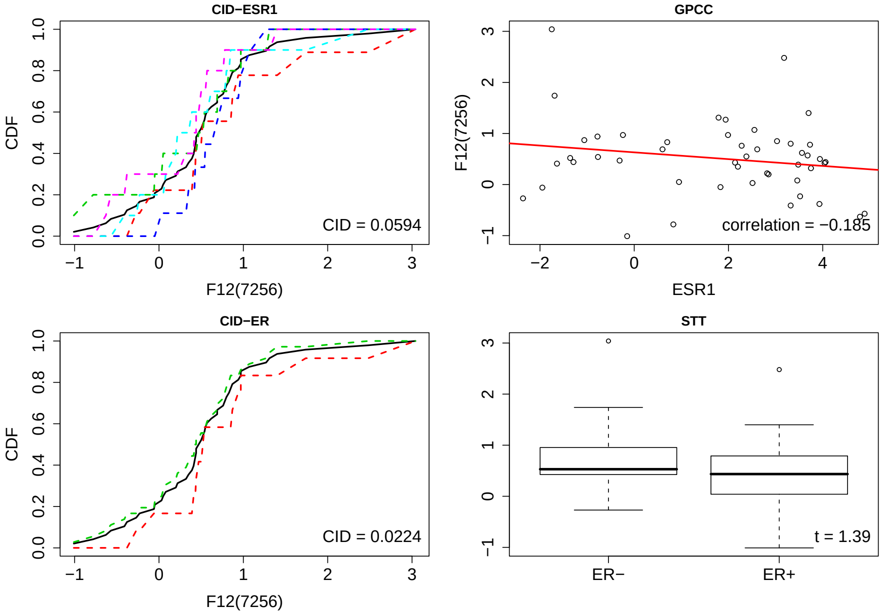
CID−ESR1
CDF
F12(7256)
CID = 0.0594
GPCC
F12(7256)
ESR1
correlation = −0.185
CID−ER
CDF
F12(7256)
CID = 0.0224
STT
ER−
ER+
t = 1.39

AGT

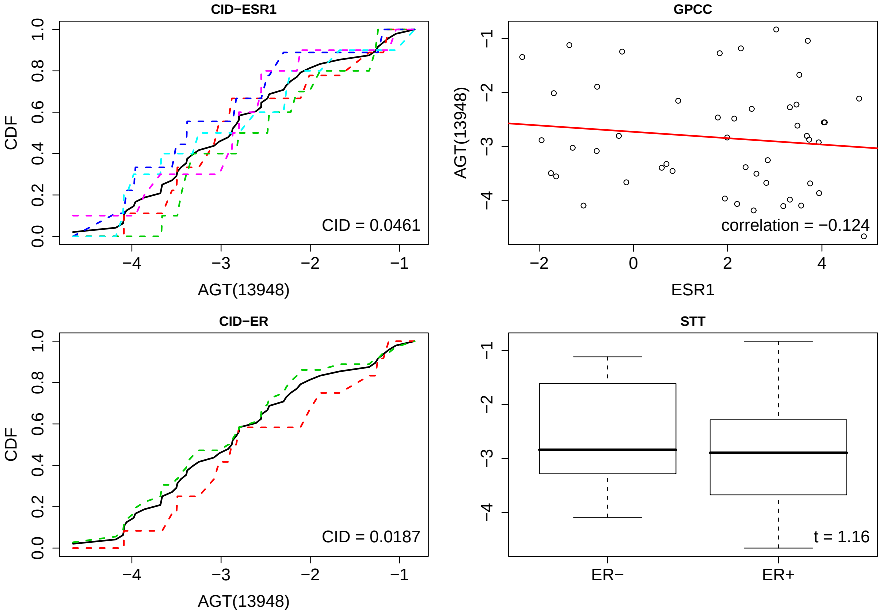
CID−ESR1
CDF
AGT(13948)
CID = 0.0461
GPCC
AGT(13948)
ESR1
correlation = −0.124
CID−ER
CDF
AGT(13948)
CID = 0.0187
STT
ER−
ER+
t = 1.16

TRIM25

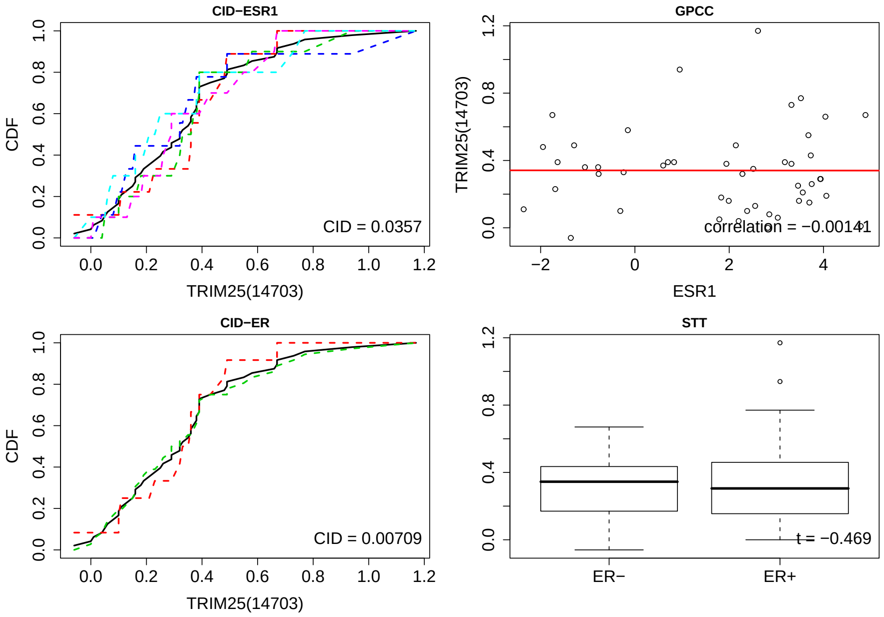

CID−ESR1
CDF
TRIM25(14703)
CID = 0.0357
GPCC
TRIM25(14703)
ESR1
correlation = −0.00141
CID−ER
CDF
TRIM25(14703)
CID = 0.00709
STT
ER−
ER+
t = −0.469
